# Molecular cloning and characterization of the porcine prostaglandin transporter (*SLCO2A1*): evaluation of its role in F4 mediated neonatal diarrhoea

**DOI:** 10.1186/1471-2156-10-64

**Published:** 2009-10-06

**Authors:** Mario Van Poucke, Vesna Melkebeek, Tim Erkens, Alex Van Zeveren, Eric Cox, Luc J Peelman

**Affiliations:** 1 Department of Nutrition, Genetics and Ethology, Faculty of Veterinary Medicine, Ghent University, Heidestraat 19, B-9820 Merelbeke, Belgium; 2 Department of Virology, Parasitology and Immunology, Faculty of Veterinary Medicine, Ghent University, Salisburylaan 133, B-9820 Merelbeke, Belgium

## Abstract

**Background:**

Because prostaglandins are involved in many (patho)physiological processes, *SLCO2A1 *was already characterized in several species in an attempt to unravel specific processes/deficiencies. Here, we describe the molecular cloning and characterization of the porcine ortholog in order to evaluate its possible involvement in F4 enterotoxigenic *E. coli *mediated neonatal diarrhoea, based on a positional candidate gene approach study.

**Results:**

Porcine *SLCO2A1 *is organized in 14 exons, containing an open reading frame of 1935 bp, encoding a 12-transmembrane organic anion cell surface transporter of 644 aa. The -388 to -5 upstream region comprises a (CpG)_48 _island containing a number of conserved promoter elements, including a TATA box. A potential alternative promoter region was found in the conserved -973 to -700 upstream region. No consensus polyadenylation signal was discovered in the 3' UTR. Repeat sequences were found in 15% of all the non coding sequences.

As expected for a multifunctional protein, a wide tissue distribution was observed. mRNA expression was found in the adrenal gland, bladder, caecum, colon (centripetal coil/centrifugal coil), diaphragm, duodenum, gallbladder, heart, ileum, jejunum, kidney, liver, longissimus dorsi muscle, lung, lymph node, mesenterium, rectum, spleen, stomach, tongue and ureter, but not in the aorta, oesophagus and pancreas.

The promoter region and the exons (including the splice sites) of *SLCO2A1 *were resequenced in 5 F4ab/ac receptor positive and 5 F4ab/ac receptor negative pigs. Two silent and 2 missense (both S → L at position 360 and 633) mutations were found, but none was associated with the F4ab/ac receptor phenotype. In addition, no phenotype associated differential mRNA expression or alternative/abberant splicing/polyadenylation was found in the jejunum.

**Conclusion:**

The molecular cloning and characterization of porcine *SLCO2A1 *not only contributes to the already existing knowledge about the transporter in general, but enables studies on porcine prostaglandin related processes/deficiencies as patient and/or model. Here we examined its possible involvement as receptor in F4 enterotoxigenic *E. coli *mediated neonatal diarrhoea. Because no phenotype associated differences could be found in the gene sequence nor in its jejunal transcription profile of F4ab/ac receptor positive/negative pigs, SLCO2A1 can most likely be excluded as receptor for F4 bacteria.

## Background

Prostaglandins are anionic fatty acid derivatives belonging to the prostanoid subclass of eicosanoids. They are synthesized by all nucleated cells (except lymphocytes) and act as autocrine/paracrine/endocrine or intracrine signal molecules by binding to their specific receptors (mostly G protein-coupled 7-transmembrane receptor family members) on the cell surface or nuclear membrane [1,2]. Prostaglandins mediate a wide range of (patho)physiological processes, including reproduction, respiration, cardiovascular homeostasis, intraocular pressure, brain activity, digestion, renal salt/water transport, bone formation, immunity, inflammation, tumorigenesis, asthma and Alzheimer's disease [3-6]. Interfering with prostaglandin production/action can have important therapeutic implications, as already shown for the clinical treatment of glaucoma and impotence, the induction of parturition/abortion and the provision of gastric protection [7].

Secreted prostaglandins have a short half-life to exert their function before their reuptake by the cell for inactivation. Although they can traverse biological membranes by passive diffusion, efficient efflux and influx is mediated by specific transporters [8]. The solute carrier organic anion transporter family, member 2A1 (SLCO2A1, alias PGT) is involved in both processes [9,10]. *SLCO2A1 *was first cloned and characterized in rat [11] and later in man [7,12], mouse [13], cow [1] and sheep [14] as a single copy gene encoding a 12-transmembrane organic anion cell surface transporter with a wide tissue distribution.

In man, *SLCO2A1 *is examined as a candidate gene for various diseases [12]. The porcine *SLCO2A1 *ortholog could be involved in F4 (alias K88) ETEC mediated neonatal diarrhoea, a common problem in pig production. F4 bacteria use their fimbriae to adhere to specific receptors on the brush borders of enterocytes of their host. This adhesion is a prerequisite for infection and promotes bacterial colonization of the small intestine. The colonizing bacteria produce enterotoxins that stimulate the secretion of water and electrolytes into the lumen of the small intestine and lead to diarrhoea and often death in neonatal pigs [15]. F4 resistance, acquired by receptor phenotype differences of the host, seems to be inherited as an autosomal recessive Mendelian trait [16]. *MUC4 *has been described as a candidate F4ab/ac receptor gene [17]. But the proposed genotypic F4 resistance associated *MUC4 *polymorphisms were not associated with total absence of adhesion of F4 bacteria to the villous brush borders [18], nor with total absence of diarrhoea [19]. These findings indicate that there is at least one other F4ab/ac receptor gene.

The search for such a receptor gene was conducted via the positional candidate gene approach. A BAC contig on porcine chromosome 13 was built by chromosome walking, covering the region around microsatellite markers Swr926 and Swc22, based on their tight linkage with F4ab/ac receptor loci [20,21]. One of the annotated genes in the contig was *SLCO2A1*, a gene producing several functionally distinct mRNAs, by using alternative promoters and/or splicing [13], and encoding prostaglandin transmembrane transporters which contain several different substrate binding sites, to which binding does not always result in substrate translocation across the membrane [7]. As it is highly expressed on intestinal epithelic cells, where prostaglandins influence intestinal fluid secretion [22] and elevated prostaglandin concentrations are shown to be correlated with diarrhoea [23], porcine *SLCO2A1 *was first characterized and then evaluated for its possible involvement in porcine F4 mediated neonatal diarrhoea.

## Results and Discussion

### Molecular characterization of porcine SLCO2A1

**Figure 1:**
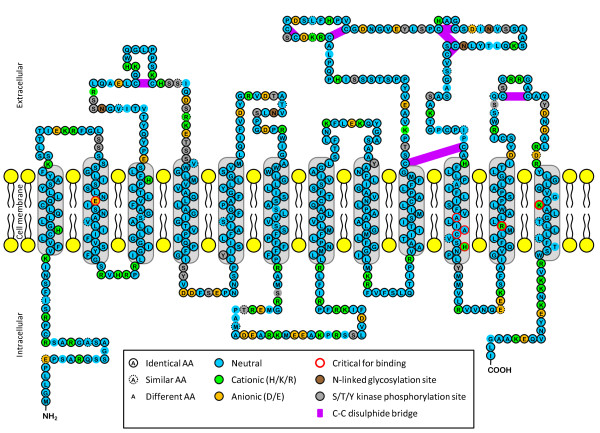
**Suggested transmembrane model of porcine SLCO2A1**. This model is based on structural and hydropathy analyses of the amino acid sequence (as performed on the bovine and ovine orthologs [1,14]) and its homology with the other described orthologs (see Additional file 2).

The porcine *SLCO2A1 *ORF consists of 1935 bp, encoding 644 aa [GenBank:NM_001123195]. This is as long as its ortholog in cow, dog and sheep, but 1 aa longer than that in man, mouse and rat. Sequence comparison shows that it is most identical with dog (Additional files 1, 2 and 3). As for the described orthologs, hydropathy and structural analyses showed that porcine SLCO2A1 contains 7 intracellular, 12 transmembrane and 6 extracellular domains (Figure 1). The presence of 10% positively and 5% negatively charged aa, makes it a cationic protein (Figure 1). Amino acid comparison with all the described mammal sequences shows that 92% of the porcine SLCO2A1 aa are identical, 4% similar and 4% different (Figure 1, Additional file 2). The 27 species specific aa are predominantly seen intracellulary (11/140 aa, of which most in the N-terminal and 4^th ^domain) and extracellulary (13/227 aa, most of which in the 2^nd ^and 5^th ^domain). Only 3 out of the 276 transmembrane aa were different (2 in last domain). The critical prostaglandin binding sites (E_78_, A_526_, A_529_, C_530_, H_533_, R_561 _and K_614_; [9,24]), C-C disulphide bridges, N-linked glycosylation sites and most of the S/T/Y kinase phosphorylation sites, all involved in the transport mechanism and predicted in cow [1] and sheep [14], were conserved in pig (Figure 1).

Porcine *SLCO2A1 *is organized in 14 exons, with the startcodon in the first and the stopcodon in the last exon. All 14 exons and adjacent intergenic/intron regions were sequenced (15,577 bp in total) [GenBank:DQ104833-46]. All exons possess consensus donor/acceptor splice sites and have exactly the same length as their bovine and canine orthologs. In comparison with the other mammals there was a maximum of 1 codon difference per exon (Table 1).

**Table 1:** Porcine genomic *SLCO2A1 *organization

**Exon No**. **Length (bp)_1_**	**Phase** **Encoding region_2_**	**Mammal length differences_2_**	**Intron No**. **Length (bp)**	**GenBank** **Acc. No**.	**Repeat sequences**	**Mutations (bp → aa)**
1 96	ID1	=	1 NA_3_	DQ104833	847-916: C-rich 1592-1721: (TG)n(GA)n(TG)n	
2 138	0 TD1-ED1-TD2	=	2 4772	DQ104834	-	C592T → S70L
3 163	0 TD2-ID2-TD3-ED2	=	3 NA_3_	DQ104835	89-109: AT-rich; 153-201: L1-SS 911-1195: L1 MD	C584A → L84L
4 228	1 ED2-TD4-ID3-TD5	=	4 796	DQ104836	-	
5 99	1 TD5-ED3	=	5 2269	DQ104836	1720-1961: PRE1e	
6 137	1 ED3-TD6-ID4	=	6 2518	DQ104838	-	
7 79	0 ID4	Mouse/Rat: -3 bp = -1 aa in I4 Sheep: -3 bp = -1 aa in I4	7 469	DQ104839	-	
8 165	1 ID4-TD7-ED4-TD8	=	8 1029	DQ104839	1193-1307: MER5B	C786T → S360L
9 190	1 TD8-ID5-TD9-ED5	=	9 1907	DQ104841	885-1106: PRE1-SS	
10 166	2 ED5	Rat: +3 bp = +1 aa in E5	10 ± 2400_3_	DQ104842	36-209: MIRb	
11 164	0 ED5-TD10-ID6	Rat: -3 bp = -1 aa in E5 Sheep: +3 bp = +1 aa in TM10	11 3481	DQ104843	1-123: CHR-1	
12 65	2 ID6-TD11	=	12 2456	DQ104844	7-169: MER5A	
13 124	1 TD11-ED6	=	13 819	DQ104845	240-342: MIRb	
14 121	2 TD12-ID7	Man: -3 bp = -1aa in I7		DQ104846	1211-1334: (CA)n 2099-2294: L3b	A398G → E633E

_1_CDS in exons; _2_ID: intracellular domain, TD: transmembrane domain, ED: extracellular domain; _3_NA: not analysed, intron 10 was only estimated by measuring the amplification product on an agarose gel.

**Figure 2:**
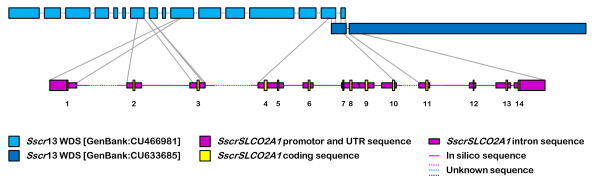
**Schematic representation of the porcine genomic *SLCO2A1 *organization and its comparison with 2 porcine WDSs**.

Porcine *SLCO2A1 *was already mapped to chromosome 13q31-q32 [21], but is not present in the pig genome sequence (assembly *Sscrofa*8 v52). However, a BLAST search in the High Throughput Genomic Sequence database revealed 2 porcine WDSs covering a big part of the gene. In the [GenBank:CU466981] sequence, containing 16 unordered pieces, homologies were found with exon 1-2, 4-6 and 10. In the [GenBank:CU633685] sequence, containing 2 unordered pieces, homologies were found with exon 5-14 (Figure 2). Because exon 2 and the 3' end of intron 2 were found in the 7^th ^unordered piece of [GenBank:CU466981], and the 5' end of intron 3 was found in the 8^th ^unordered piece, the gap between piece 7 and 8 could be closed with our [GenBank:DQ104835] sequence, containing exon 3, and the exact length of intron 2 could be calculated. The 10^th ^unordered piece, containing exon 1, should be replaced before the joined pieces 7-8. Because of the gaps, no intron lengths could be calculated from intron 1 and 3 (estimated in man as 48 and 18 kb resp.; [12]). Since pieces 15 (containing exon 4-6) and 16 (containing exon 10) of sequence [GenBank:CU466981] overlap with the first piece of sequence [GenBank:CU633685] (containing exons 5-10), the gap between pieces 15 and 16 could be filled and the lengths of introns 4-9 could be calculated. Exons 11-14 were found in the 2^nd ^piece of [GenBank:CU466981] and as a result also the exact intron lengths of introns 11-13 could be calculated. Both pieces of [GenBank:CU633685] are correctly ordered and orientated, and the gap between them, containing a part of intron 10, should be around 100 bp based on the estimated length of intron 10 after PCR with primers F33/R34 and gelelectrophoresis (Table 1).

**Figure 3:**
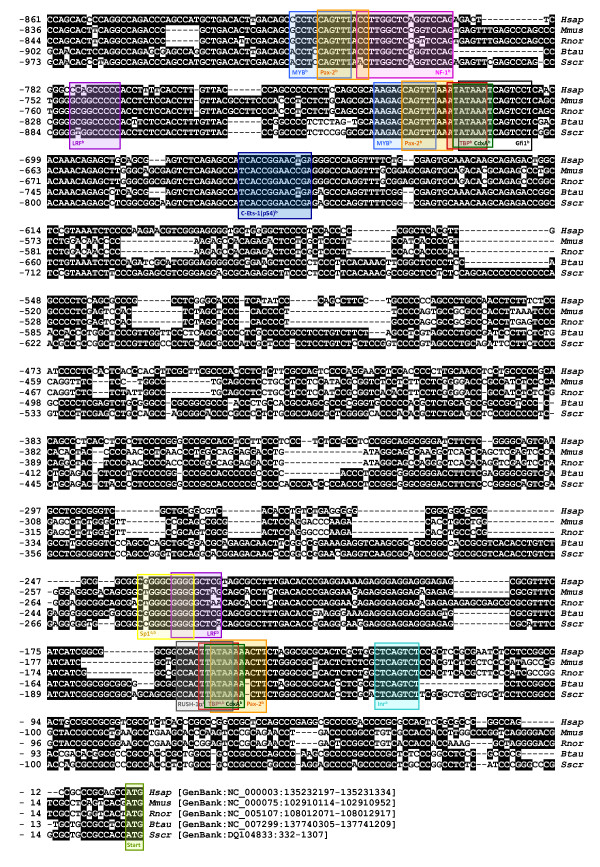
***SLCO2A1 *promotor sequence alignment in man (*Hsap*), mouse (*Mmus*), rat (*Rnor*), cow (*Btau*) and pig (*Sscr*)**. Conserved sequences are shaded in black. In silico detected motifs (^a^described by Lu and Schuster [12]; ^b ^discovered with ConTra [35]) are indicated in coloured boxes.

About 1300 bp upstream of the ATG startcodon were sequenced and compared with the orthologous genomic sequence in man, mouse, rat and cow (Figure 3). The -388 to -5 region comprises a CpG island with 48 CpGs. It contains a number of conserved promoter elements, such as a transcription initiation (Inr), TATA box (TBP), Pax-2, CdxA, RUSH-1α, LRF and Sp1 consensus site and was already described as the promoter region in man [12]. It also contains a region (-355 to -260) that seems to be specific for primates (man), rodents (mouse and rat) and cetartiodactyls (cow and pig). The -700 to -389 region is less conserved, containing a pig specific insertion (-638 tot -624: AGCACCCCCCCCCCC) and a C-rich region (-458 to -389). Remarkable is the conservation of the -973 to -700 region. It contains conserved TATA box (TBP), CdxA, MYB, Pax-2, NF-1, Gfi1, LRF and C-Ets-1(p54) consensus sites, and must be considered as a possible alternative promoter region. No consensus polyadenylation signal was discovered in the 3' UTR, as in man [12]. Fifteen percent of all the non coding sequences were repeat sequences, including a (TG)_14_(GA)_39_(GC)_2_(GA)_2_(GC)_2_(GT)_2_(GC)_5_-repeat in the beginning of intron 1 and a CAGA(CA)_19_C_4_GCTGCAGA(CA)_9_C_4_GCTGCAGA(CA)_21_-repeat in the 3' UTR (Table 1).

### Porcine SLCO2A1 transcription profiling

**Figure 4:**
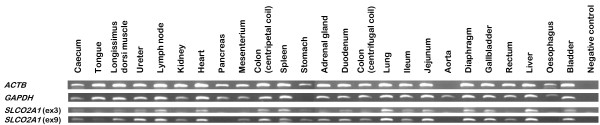
**Transcription profiling of *SLCO2A1 *in 25 porcine tissues by RT-PCR**. *ACTB *and *GAPDH *were used as reference genes. *SLCO2A1 *mRNA transcription was analyzed with amplicons from exon 3 and 9.

DNA-free RNA was isolated out of 25 different porcine tissues and reverse transcribed into cDNA. PCR was performed with *ACTB *and *GAPDH *as positive controls (Figure 4). *ACTB *mRNA was present in all tissues except in the aorta. This is in agreement with the expression data provided by Unigene, except for the fact that we could demonstrate *ACTB *mRNA transcription in pancreas. *GAPDH *mRNA was present in all tissues except in stomach. In contrast with the data provided by Unigene, we could not detect *GAPDH *mRNA in stomach, but we could detect it in aorta, bladder, oesophagus and pancreas. These data show that all our samples contain cDNA, but that care should be taken when using a single reference gene as positive control in transcription profiling, even for RT-PCR.

Porcine *SLCO2A1 *mRNA expression was evaluated in all 25 tissues with 2 different primer pairs, amplifying fragments of resp. exon 3 and 9 (Figure 4). Transcription was seen in the adrenal gland, bladder, caecum, colon (centripetal coil/centrifugal coil), diaphragm, duodenum, gallbladder, heart, ileum, jejunum, kidney, liver, longissimus dorsi muscle, lung, lymph node, mesenterium, rectum, spleen, stomach, tongue and ureter. This wide tissue distribution was expected since prostaglandins mediate a wide range of (patho)physiological processes and it was also observed in man, mouse, rat and cow [1,8]. No *SLCO2A1 *mRNA was detected in the aorta, oesophagus and pancreas.

### Evaluation of SLCO2A1 involvement in porcine F4 mediated neonatal diarrhoea

Based on the positional candidate gene approach study by Van Poucke and co-workers [21] and taking into account its functional characteristics and its abundant mRNA expression in the porcine jejunum intestine, *SLCO2A1 *was evaluated for its possible involvement in porcine F4 mediated neonatal diarrhoea.

A region of 1300 bp upstream of the startcodon, the complete ORF (1935 bp), all splice sites and a region of 1900 bp downstream of the stopcodon were resequenced in 5 F4ab/ac receptor positive and 5 F4ab/ac receptor negative pigs, all phenotyped via the *in vitro *villous adhesion test as described by Rasschaert and co-workers [18], in order to identify phenotype associated *SLCO2A1 *polymorphisms. As the F4 ab/ac receptor phenotype is monogenic or oligogenic, it is to be expected of a mutation responsible for the phenotypes to be present in one group and not in the other, or at least show a significant distribution difference that can easily be seen in a small number of animals. This screening identified 4 *SLCO2A1 *mutations (Table 1), 2 silent and 2 missense (both S → L) mutations. The 2 silent mutations were identified in heterozygous state in only 1 F4ab/ac receptor positive animal. The 2 missense mutations were exclusively found in heterozygous state in both F4ab/ac receptor positive and F4ab/ac receptor negative animals. Based on these observations, it is clear that neither of the polymorphisms is associated with the F4ab/ac receptor phenotype.

Because of the big phenotype difference between sensitive or resistant (binding or not), we additionally investigated the *SLCO2A1 *mRNA expression in the jejunum of 8 pigs with a different phenotype (3 strong F4ab/ac receptor positive, 2 weak F4ab/ac receptor positive and 3 F4ab/ac receptor negative animals), based on the *in vitro *villous adhesion test [18]. The RT-PCR assay was performed with 5 different exon spanning primer pairs covering the whole *SLCO2A1 *transcript (except for exon 1) in order to simultaneously investigate any alternative/abberant splicing/polyadenylation. However, all pigs displayed a high *SLCO2A1 *mRNA expression in the jejunum and no phenotype associated alternative/abberant splicing/polyadenylation was observed (Additional file 4).

## Conclusion

Because of its role in many (patho)physiological processes, it is necessary to know the molecular structure of *SLCO2A1 *as a basis for unraveling its function. In this paper we described the molecular structure of the porcine ortholog. The analysis not only contributes to the already existing knowledge about *SLCO2A1 *in general, but can also be used in the assembly/annotation of the porcine genome and in future studies on porcine SLCO2A1 related processes/deficiencies as a patient or as a biomedical model [25]. Here we examined its possible involvement in F4 ETEC mediated neonatal diarrhoea, based on a positional candidate gene approach study. As no phenotype associated polymorphisms could be found in the promotor region and all exons (including the splice sites) of *SLCO2A1*, and no phenotype associated differential *SLCO2A1 *mRNA expression or alternative/abberant splicing/polyadenylation could be detected in the porcine jejunum, SLCO2A1 can most likely be excluded as receptor for F4 ETEC.

## Methods

### Primer design, PCR and sequencing

Primers were designed using Primer3 [26] according to the acceptable primer design guidelines and selected taking into account the absence of secondary structures (mfold, [27]) and repeat sequences (RepeatMasker, [28]). Primer/amplicon characteristics are listed in Additional file 5.

Default PCRs were performed in a total volume of 10 μl on 100 ng genomic DNA, 20 ng BAC DNA or cDNA (an equivalent of 5 ng RNA) as a template with 500 nM of each primer, 200 μM of each dNTP, 2 mM MgCl_2 _and 0.5 U FastStart Taq DNA Polymerase (Roche). Default PCR programs for PCR amplicons of <500/500><1000/>1000 bp consisted of an initial 4-min denaturation step at 95°C, followed by 30 cycles of 15/30/45 s denaturation at 95°C, 15/30/45 s annealing at 61°C and 30/60/90 s elongation at 72°C, and a final 7-min elongation step at 72°C. PCR conditions different from default are mentioned in Additional file 5. Sequencing reactions were performed with the BigDye Terminator v3.1 Cycle Sequencing Kit (Applied Biosystems) and after purification with magnetic beads (Agencourt) analyzed on a 3730xl DNA Analyzer (Applied Biosystems), according to the manufacturers' instructions.

### Template preparation

Porcine blood collection, storage and DNA isolation was performed as described by Van Poucke and co-workers [29]. The screening, annotation and DNA isolation of BAC clone 884H1 was described by Van Poucke and co-workers [21]. Fresh samples of 25 different tissues (adrenal gland, aorta, bladder, caecum, colon (centripetal coil/centrifugal coil), diaphragm, duodenum, gallbladder, heart, ileum, jejunum, kidney, liver, longissimus dorsi muscle, lung, lymph node, mesenterium, oesophagus, pancreas, rectum, spleen, stomach, tongue and ureter) were taken from one pig immediately after slaughtering and kept in liquid nitrogen. They were subsequently crushed in a mortar and 100 mg was used to isolate 1-10 μg of total RNA with 1 ml Total RNA Isolation Reaction (Abgene). Possible traces of genomic DNA were removed by a RQ1 DNase digest (Promega), followed by a YM-100 microcon purification step (Millipore). This was verified by a minus RT control using intron-spanning primers of porcine *TOP2B *[30]. One μg of DNA-free total RNA was converted into cDNA by using the iScript cDNA Synthesis Kit (Bio-Rad). PCRs with primers amplifying *ACTB *and *GAPDH *were used to control for the cDNA synthesis [31].

The jejunum samples of 8 pigs with a different F4ab/ac receptor phenotype (3 strong F4ab/ac receptor positive, 2 weak F4ab/ac receptor positive and 3 F4ab/ac receptor negative animals), assessed via the *in vitro *villous adhesion test [18], were treated in a similar way, except that the RNA isolation was carried out using the Aurum Total RNA Fatty and Fibrous Tissue Kit (Bio-Rad) and the cDNA synthesis with the ImProm-II Reverse Transcriptase Kit (Promega). Experimental procedures and animal management procedures were undertaken in accordance with the requirements of the animal care and ethics committee of the Faculty of Veterinary Medicine, Ghent University, Belgium (EC2005/65).

### Porcine SLCO2A1 sequencing and annotation

Three overlapping porcine *SLCO2A1 *cDNA amplicons, covering exon 2 to exon 14, were generated with cDNA synthesized from RNA isolated from porcine jejunum as a template, using primers F1/R1-F3/R3 (all based on human *SLCO2A1 *[GenBank:U70867] because at the time of sequencing no porcine genomic sequences were available yet) and sequenced with all respective PCR primers as sequence primers. The 5' and 3' end of the cDNA was amplified by using the GeneRacer Kit (Invitrogen). Primers R4 and R5 were based on the *de novo *porcine exon 3 *SLCO2A1 *sequence and used to identify the 5' end of the porcine *SLCO2A1 *transcript. Primers F6 and F7 were based on an anonymous porcine EST [GenBank:CF788195], that showed 82% sequence identity with the 3' UTR of human *SLCO2A1*, and was used to identify the 3' end of the porcine *SLCO2A1 *transcript. The rest of the sequence was identified by direct sequencing of porcine BAC 884H1, previously isolated, mapped and shown to contain *SLCO2A1 *by Van Poucke and co-workers [21], with primers based on the *de novo *porcine *SLCO2A1 *sequence. The promoter region was sequenced by primer walking with primers F8 and R9-R11. The gap between the coding sequence in exon 14 and the 3' end was filled by primer walking with primers F24-F25 and R25-R26. All exon-intron bounderies were determined with primers F1, F8, F13, F15-F23 and R2-R3, R12-R19, R21, R23. Sequence database searches were performed with NCBI software (BLAST tool and Nucleotide, Gene and UniGene databases; [32]), sequence assemblies with CAP [33], multiple sequence alignments with ClustalW [34] and the identification of conserved promoter elements with ConTra [35]. Resequencing of the promoter region and all exons with splice sites was performed via direct sequencing of PCR amplicons (with primers F9/R9-F11/R11, F15/R15, F22/R22 and F24/R24-F36/R36 as both PCR and sequence primers), generated from genomic DNA isolated from blood from 5 F4ab/ac receptor positive and 5 F4ab/ac receptor negative pigs, all phenotyped via the *in vitro *villous adhesion test as described by Rasschaert and co-workers [18].

### Porcine SLCO2A1 transcription profiling

Porcine *SLCO2A1 *mRNA expression was evaluated in all 25 tissues with primers F13/R13 and F19/R19. Potential differential *SLCO2A1 *mRNA expression and/or alternative/abberant splicing/polyadenylation in the jejunum of 8 pigs with a different phenotype (3 strong F4ab/ac receptor positive, 2 weak F4ab/ac receptor positive and 3 F4ab/ac receptor negative animals), based on the *in vitro *villous adhesion test, was investigated by RT-PCR with primers F18/R18, F26/R26 and F37/R37-F39/R39.

## List of abbreviations

aa: amino acids; *ACTB*: gene encoding actin, beta; BAC: bacterial artificial chromosome; bp: base pairs; ETEC: enterotoxigenic *Escherichia coli*; *GAPDH*: gene encoding glyceraldehyde-3-phosphate dehydrogenase; *MUC4*: gene encoding mucin 4, cell surface associated; ORF: open reading frame; RT-PCR: reverse transcription - polymerase chain reaction; *SLCO2A1*: gene encoding solute carrier organic anion transporter family, member 2A1; *TOP2B*: gene encoding topoisomerase (DNA) II beta 180 kDa; UTR: untranslated region; WDS: working draft sequence.

## Authors' contributions

MVP designed, coordinated and carried out most of the work, and drafted this manuscript. VM assisted in sample collection and phenotyping. TE assisted in sample collection. EC supervised the phenotyping. AVZ and LJP designed the project. All authors read and approved the final manuscript.

## Supplementary Material

Additional file 1 **Nucleic acid sequence alignment between porcine *SLCO2A1 *and its published orthologs in man, mouse, rat, cow, dog and sheep**. Comparative *SLCO2A1 *nucleic acid sequence alignment with indication of the conserved sequences, the exon bounderies, the translated porcine amino acid sequence and the predicted protein domains. Click here for file

Additional file 2 **Amino acid sequence alignment between porcine SLCO2A1 and its published orthologs in man, mouse, rat, cow, dog and sheep**. Comparative *SLCO2A1 *amino acid sequence alignment with indication of the conserved sequences and the predicted protein domains. Click here for file

Additional file 3 **Porcine *SLCO2A1 *sequence comparison**. Overview in percentage of the porcine *SLCO2A1 *nucleic acid and amino acid sequence homologies with its published orthologs in man, mouse, rat, cow, dog and sheep. Click here for file

Additional file 4 ***SLCO2A1 *mRNA expression profiling in the jejunum of 8 pigs with a different F4ab/ac receptor phenotype by RT-PCR**. Agarose gels showing 5 overlapping (covering the complete transcript) jejunal *SLCO2A1 *RT-PCR products of 8 receptor positive/negative pigs. Click here for file

Additional file 5 **Primer names/sequences/positions, amplicon sizes and PCR conditions**. Table including all information needed to perform all PCR/sequencing reactions described in this paper. Click here for file
