# Supplementary material for: Molecular cloning and characterization of the porcine prostaglandin transporter (SLCO2A1): evaluation of its role in F4 mediated neonatal diarrhoea
Source: BMC Genet. 2009 Oct 6;10:64. doi: 10.1186/1471-2156-10-64 (PMC2763009; doi:10.1186/1471-2156-10-64)
Supplement: Additional file 1 — Nucleic acid sequence alignment between porcine SLCO2A1 and its published orthologs in man, mouse, rat, cow, dog and sheep. Comparative SLCO2A1 nucleic acid sequence alignment with indication of the conserved sequences, the exon bounderies, the translated porcine amino acid sequence and the predicted protein domains. [file 1471-2156-10-64-S1.PDF]

Additional file 1. Nucleic acid sequence alignment between porcine (*Sscr*) *SLCO2A1* [GenBank:NM_001123195] and its published orthologs in man (*Hsap*) [Genbank:NM_005630], mouse (*Mmus*) [Genbank:NM_033314], rat (*Rnor*) [Genbank:NM_022667], cow (*Btau*) [Genbank:NM_174829], dog (*Cfam*) [Genbank:NM_001011558] and sheep (*Oari*) [Genbank:DQ026455]. Conserved nucleic acids are in black. Exon bounderies are indicated above the sequence alignment. The translated porcine amino acid sequence and the predicted intracellular (ID), transmembrane (TD) and extracellular (ED) domains are indicated below the sequence alignment.

```
HsapSLCO2A1   ATGGGGCTCCTGCCCAAGCTCGGCGTGTCCCAGGGCAGCGACACCTCTACTAGCCGAGCC  60
MmusSLCO2A1   ATGGGGCTCCTGCCCAAGCCCGGAGCACGCCAGGGCAGCGGCACCTCCTCGGTCCCAGCC  60
RnorSLCO2A1   ATGGGGCTCCTGCTCAAGCCTGGAGCGCGCCAGGGCAGCGGCACCTCCTCGGTCCCAGAC  60
BtauSLCO2A1   ATGGGGTTCCTGCCCAAGCCCGGCGCGCGCCAGGGTAGCGCCGCCTCTACTGGCCAAGCC  60
CfamSLCO2A1   ATGGGGCTCCTGCCCAAGCCCGACGCGCGCCGCGGCAGCGACGCCTCTCCCGGCCGGCCC  60
OariSLCO2A1   ATGGGGCTCCTGCCCAAGCTCGGCGCGCGCCAGGGTAGCGCCGCCTCTGCGGGCCGAGCC  60
SscrSLCO2A1   ATGGGGCTCCTGCCTGAGCCCAGCGCACGCCAGAGCAGCGGCGCCTCTGCGGGCCGAGCC  60
               M  G  L  L  P  E  P  S  A  R  Q  S  S  G  A  S  A  G  R  A

                                                 EX1><EX2
HsapSLCO2A1   GGCCGCTGTGCCCGCTCGGTCTTCGGCAACATTAAGGTGTTTGTGCTCTGCCAAGGCCTC  120
MmusSLCO2A1   AGACGTTGCTCCCGCTCGGTCTTCAACAACATTAAGGTCTTCGTGCTTTGTCATGGCCTC  120
RnorSLCO2A1   AGACGTTGTCCCCGCTCCGTCTTCAGCAACATTAAGGTATTTGTTCTTTGCCATGGCCTG  120
BtauSLCO2A1   GGCTGCAGTCCCCGCTCTATCTTCAGCAACATTAAGGTGTTCGTGCTGTGCCATGGCCTC  120
CfamSLCO2A1   CGCCGCTGTCCGCGCTCCGTCTTCAGCAACATTAAGGTGTTCGTGCTCTGCCACGGCCTC  120
OariSLCO2A1   GGCTGCAATCCCCGCTCTATCTTCAGCAACATTAAGGTGTTTGTGCTGTGCCATGGCCTC  120
SscrSLCO2A1   AGCCGCTGTCCCCGCTCTATCTTCAGCAACATTAAGGTGTTCGTGCTCTGCCATGGCCTC  120
               S  R  C  P  R  S  I  F  S  N  I  K  V  F  V  L  C  H  G  L
                                                 ID1><TD1

HsapSLCO2A1   CTGCAGCTCTGCCAACTCCTGTACAGCGCCTACTTCAAGAGCAGCCTCACCACCATTGAG  180
MmusSLCO2A1   CTACAGCTCTGCCAGTTGCTCTACAGTGCCTACTTCAAGAGCAGCCTCACCACTATCGAG  180
RnorSLCO2A1   CTACAGCTCTGCCAGCTGCTCTACAGCGCCTACTTCAAGAGCAGTCTCACCACAATCGAG  180
BtauSLCO2A1   CTGCAGCTCTGCCAGCTCCTGTATAGTGCCTACTTCAGGAGCAGCCTCACCACCATCGAG  180
CfamSLCO2A1   CTGCAGCTGTGCCAGCTCCTGCACAGCGCCTACTTCAAGAGCAGCCTCACCACCATCGAG  180
OariSLCO2A1   TTGCAGCTCTGCCAGCTCCTGTATAGTGCCTACTTTAAGAGCAGCCTCACCACCATCGAG  180
SscrSLCO2A1   CTGCAGCTCTGCCAGCTCCTGTACAGCGCCTACTTCAAGAGCAGCCTCACCACCATCGAG  180
               L  Q  L  C  Q  L  L  Y  S  A  Y  F  K  S  S  L  T  T  I  E
                                                 TD1><ED1

                                                                EX2><EX3
HsapSLCO2A1   AAGCGCTTTGGGCTCTCCAGTTCTTCATCGGGTCTCATTTCCAGCTTGAATGAGATCAGC  240
MmusSLCO2A1   AAGCGCTTTGGGCTCTCCAGTTCTTCCTCCGGTCTCATCTCCAGTTTGAATGAGATCAGC  240
RnorSLCO2A1   AAGCGCTTTGGGCTCTCCAGCTCTTCCTCTGGTCTCATCTCCAGTTTGAATGAGATCAGC  240
BtauSLCO2A1   AAGCGGTTTGGCCTCTCCAGTTCTTCGTCAGGCTTCATCTCCAGCTTGAATGAGATCAGC  240
CfamSLCO2A1   AAGCGCTTTGGCCTCTCCAGTTCCTCCTCAGGTCTCATCTCCAGCTTGAATGAGATCAGC  240
OariSLCO2A1   AAGCGGTTTGGCCTCTCCAGTTCTTCCTCAGGTTTCATCTCCAGCTTGAATGAGATTAGC  240
SscrSLCO2A1   AAGCGCTTTGGCCTCTCCAGTTCTTCCTCGGGCCTCATTTCCAGCTTGAATGAGATCAGC  240
               K  R  F  G  L  S  S  S  S  S  G  L  I  S  S  L  N  E  I  S
                                     ED1><TD2
```

```
HsapSLCO2A1   AATGCCATCCTCATCATCTTTGTCAGCTACTTTGGCAGCCGGGTGCACCGTCCACGTCTG  300
MmusSLCO2A1   AACGCTATCCTCATCATCTTCGTCAGCTACTTCGGCAGCCGGGTGAACCGCCCACGGATG  300
RnorSLCO2A1   AACGCTACCCTCATCATCTTCATTAGCTACTTCGGCAGCCGGGTCAACCGCCCACGGATG  300
BtauSLCO2A1   AACGCCGTCCTCATCATCTTCGTCAGCTACTTTGGCAGCCGGGTGCACCGTCCAAGGCTG  300
CfamSLCO2A1   AATGCCATCCTCATCATCTTTGTCAGCTACTTTGGCAGCCGGGTGCACCGTCCACGGCTG  300
OariSLCO2A1   AACGCCATCCTCATCATCTTCGTCAGCTACTTTGGCAGCCGGGTCCACCGTCCAAGGCTG  300
SscrSLCO2A1   AACGCCGTCCTCATCATCTTTGTCAGCTACTTTGGCAGCCGCGTGCACCGTCCGAGGCTG  300
               N  A  V  L  I  I  F  V  S  Y  F  G  S  R  V  H  R  P  R  L
                                             TD2><ID2          ID2><TD3

HsapSLCO2A1   ATTGGCATCGGAGGTCTCTTCCTGGCTGCAGGTGCCTTCATCCTCACCCTCCCACACTTC  360
MmusSLCO2A1   ATCGGCATAGGGGGTCTCCTCCTGGCTGCAGGGGCCTTTGTCCTCACCCTCCCACACTTC  360
RnorSLCO2A1   ATTGGCATAGGGGGTCTCCTCCTGGCTGCAGGGGCCTTTGTCCTCACCCTCCCACACTTC  360
BtauSLCO2A1   ATTGGCATTGGGGGTCTTCTGCTAGCTTTGGGGGCCTTTATCCTCACCCTCCCACACTTC  360
CfamSLCO2A1   ATTGGCATTGGGGGTCTCCTCCTAGCTTCGGGTGCCTTCATCCTCACCCTCCCACACTTC  360
OariSLCO2A1   ATTGGCATTGGGGGTCTTCTGCTAGCTTTGGGGGCCTTTGTCCTCACCCTCCCACACTTC  360
SscrSLCO2A1   ATTGGCATTGGGGGTCTCCTCCTGGCTTCTGGGGCCTTCGTCCTCACCCTCCCGCACTTC  360
               I  G  I  G  G  L  L  L  A  S  G  A  F  V  L  T  L  P  H  F

                                               EX3><EX4
HsapSLCO2A1   CTCTCCGAGCCCTACCAGTACACCTTGGCCAGCACTGGGAACAACAGCCGCTTGCAGGCC  420
MmusSLCO2A1   CTGTCAGAACCCTATCAGTACGCGTCGACAACTGCTGGAAACAGCAGCCACTTTCAGACC  420
RnorSLCO2A1   CTGTCAGAGCCCTATCAATACACCTCGACCACGGATGGAAACAGGAGCAGCTTTCAGACT  420
BtauSLCO2A1   CTCTCAGAGCCTTACCAATACACCAAGACCATCATGGGGAACAGCAGCCACCTCCAGACC  420
CfamSLCO2A1   CTGTCAGAGCCCTACCAGTACACCTTGGCCAGCGTTGGGAACAGCAGCCACTTCCAAGCT  420
OariSLCO2A1   CTCTCAGAGCCTTACCAATACACCAAGACCATCATGGGGAACAGCAGCCACCTCCAGACC  420
SscrSLCO2A1   CTCTCCGAGCCGTACCAATACACCGTGACCATCGTGGGGAACAGCAGCCGCCTCCAGGCT  420
               L  S  E  P  Y  Q  Y  T  V  T  I  V  G  N  S  S  R  L  Q  A
                TD3><ED2

HsapSLCO2A1   GAGCTCTGCCAGAAGCATTGGCAGGACCTGCCTCCCAGTAAGTGCCACAGCACCACCCAG  480
MmusSLCO2A1   GACCTCTGTCAGAAGCATTTGCCAGGCCTGCTCCCCAGTAAGTGCCACAGCACCGTGCCC  480
RnorSLCO2A1   GACCTCTGTCAGAAGCATTTCGGAGCCCTGCCCCCCAGTAAGTGCCATAGCACCGTGCCA  480
BtauSLCO2A1   GAGCTCTGTCAGAAATCCTGGCAGGGCCTGCCCCCCAGCAAGTGCCACAGCAGCCCCCAG  480
CfamSLCO2A1   GAGCTCTGTCAGAAGCACTGGCAGGACCTTCCCCCCAGTAAGTGCCATAGCACCACCCAG  480
OariSLCO2A1   GAGCTCTGTCAGAAAGCCTGGCAGGGCCTGCCCCCCAGCAAGTGCCACAGCAGCCCCCAA  480
SscrSLCO2A1   GAGCTCTGTCAGAAACACTGGCAGGGCCTGCCCCCCAGCAAGTGCCACAGCAGCATCCAG  480
               E  L  C  Q  K  H  W  Q  G  L  P  P  S  K  C  H  S  S  I  Q

HsapSLCO2A1   AACCCCCAGAAGGAGACCAGCAGCATGTGGGGCCTGATGGTGGTTGCCCAGCTGCTGGCT  540
MmusSLCO2A1   GATACCCAGAAGGAGACCAGCAGCATGTGGAGCCTGATGGTGGTCGCTCAGCTGCTGGCC  540
RnorSLCO2A1   GATACCCACAAGGAGACCAGCAGCCTGTGGGGCCTGATGGTGGTTGCTCAACTACTGGCC  540
BtauSLCO2A1   GACTCTCAGAAGGAGACCAGCAGCATGTGGGGCCTGATGGTGATCGCCCAGCTACTGGCA  540
CfamSLCO2A1   GACTCCCGGAAGGAGACCAGCAGCATGTGGGGTCTCATGGTGGTCGCCCAGCTGCTGGCA  540
OariSLCO2A1   GACTCTCGGAAGGAGACCAGCAGCATGTGGGGCCTGATGGTGATCGCCCAGCTACTGGCA  540
SscrSLCO2A1   GACTCCCGGAAGGAGACCAGCAGCGTGTGGGGTCTCATGGTGGTGGCCCAGCTGCTGGCA  540
               D  S  R  K  E  T  S  S  V  W  G  L  M  V  V  A  Q  L  L  A
                             ED2><TD4
```

```
HsapSLCO2A1  GGCATCGGGACAGTGCCTATTCAGCCATTTGGGATCTCCTATGTGGATGACTTCTCAGAG  600
MmusSLCO2A1  GGCGTTGGGACGGTGCCCATTCAGCCATTTGGGATCTCCTATGTGGACGACTTTGCAGAG  600
RnorSLCO2A1  GGCATTGGGACAGTGCCCATCCAGCCCTTTGGGATCTCCTACGTGGATGACTTTGCCGAG  600
BtauSLCO2A1  GGCATCGGCACGGTGCCCATCCAGCCATTTGGGATCTCCTACGTGGATGACTTCTCGGAG  600
CfamSLCO2A1  GGCGTCGGGACGGTGCCCATTCAGCCATTCGGAATCTCCTACGTGGATGACTTCTCTGAG  600
OariSLCO2A1  GGCATCGGCACGGTGCCCATCCAGCCATTTGGGATCTCCTACGTGGATGACTTCTCGGAG  600
SscrSLCO2A1  GGCATTGGCACGGTGCCCATCCAGCCGTTTGGAATCTCCTATGTGGATGACTTCTCAGAG  600
              G  I  G  T  V  P  I  Q  P  F  G  I  S  Y  V  D  D  F  S  E
                                                 TD4><ID3
```

```
                           EX4><EX5
HsapSLCO2A1  CCCAGCAACTCGCCCCTGTACATCTCCATCTTATTTGCCATCTCTGTATTTGGACCGGCT  660
MmusSLCO2A1  CCTACCAACTCGCCTCTGTATATCTCCATCTTATTTGCTATCGCCGTGTTTGGGCCGGCT  660
RnorSLCO2A1  CCTACCAACTCACCTCTGTATATCTCCATCCTATTCGCCATCGCTGTGTTCGGACCGGCT  660
BtauSLCO2A1  CCCAACAACTCGCCCCTGTACATCTCCATCCTGTTTGCCATCGCCGTGTTTGGACCAGCT  660
CfamSLCO2A1  CCCAACAACTCACCCCTGTACATCTCCATCTTATTTGCCATCTCTGTGTTTGGACCGGCC  660
OariSLCO2A1  CCCAACAACTCGCCCCTGTACATCTCCATCCTGTTTGCCATCGCCGTGTTTGGACCAGCT  660
SscrSLCO2A1  CCCAACAACTCGCCCCTGTACATCTCCATCTTATTTGCCATCTCTGTATTCGGACCGGCT  660
              P  N  N  S  P  L  Y  I  S  I  L  F  A  I  S  V  F  G  P  A
                    ID3><TD5
```

```
HsapSLCO2A1  TTCGGGTACCTGCTGGGCTCTATCATGCTGCAGATCTTTGTGGACTATGGCAGGGTCAAC  720
MmusSLCO2A1  TTCGGGTACCTGCTGGGCTCAGTCATGCTGAGGATCTTCGTGGACTACGGCAGAGTGGAC  720
RnorSLCO2A1  TTCGGGTACCTGCTGGGCTCAGTCATGCTGAGAATCTTCGTGGACTACGGCAGAGTGGAC  720
BtauSLCO2A1  TTCGGGTACCTGCTGGGCTCGGTCATGCTGCAGATCTTTGTAGACTATGGCAGGGTGGAC  720
CfamSLCO2A1  TTCGGATACCTGCTGGGCTCCGTCATGCTGCAGATCTTTGTGGACTATGGCCGAGTGGAC  720
OariSLCO2A1  TTCGGGTACCTGCTGGGCTCAGTCATGCTGCAGATCTTTGTAGACTATGGCAGGGTGGAC  720
SscrSLCO2A1  TTCGGCTACCTGCTGGGCTCGGTCATGCTGCAGATCTTTGTGGACTACGGCAGGGTGGAC  720
              F  G  Y  L  L  G  S  V  M  L  Q  I  F  V  D  Y  G  R  V  D
                                TD5><ED3
```

```
             EX5><EX6
HsapSLCO2A1  ACAGCTGCAGTTAACTTGGTCCCGGGTGACCCCCGATGGATTGGAGCCTGGTGGCTAGGC  780
MmusSLCO2A1  ACGGCTACAGTGAACCTGAGCCCAGGTGACCCTCGGTGGATCGGAGCCTGGTGGCTGGGC  780
RnorSLCO2A1  ACTGCTACCGTAAACCTGAGCCCAGGTGACCCTCGGTGGATTGGAGCCTGGTGGCTGGGC  780
BtauSLCO2A1  ACAGCTTCGGTTAACTTGAGCCCTGGAGACCCCCGATGGATAGGAGCCTGGTGGTTGGGC  780
CfamSLCO2A1  ACAGCTGCGGTTAACCTGAGCCCTGGAGATCCCCGGTGGATTGGAGCATGGTGGCTGGGC  780
OariSLCO2A1  ACAGCTTCTGTTAACTTGAGCCCTGGAGACCCCCGATGGATAGGAGCCTGGTGGTTGGGC  780
SscrSLCO2A1  ACAGCTACGGTTAACTTAAGCCCTGGAGACCCTCGGTGGATAGGAGCCTGGTGGCTGGGC  780
              T  A  T  V  N  L  S  P  G  D  P  R  W  I  G  A  W  W  L  G
                                                         ED3><TD6
```

```
HsapSLCO2A1  CTGCTCATTTCTTCAGCTTTATTGGTTCTCACCTCTTTCCCCTTTTTTTTCTTCCCTCGA  840
MmusSLCO2A1  CTGCTCATCTCCTCAGGCTTCTTGATCGTCACCTCTTTGCCCTTTTTCTTCTTTCCCCGA  840
RnorSLCO2A1  CTGCTCATCTCCTCAGGCTTCTTGATTGTCACCTCTTTGCCCTTCTTTTTCTTTCCCCGA  840
BtauSLCO2A1  CTGCTCATTTCCTCTGCCTGTTTGGTAGTCACTTCTTTCCCCTTCTTCTTCTTTCCTCGA  840
CfamSLCO2A1  CTGCTCATCTCCTCAGCCTCCTTGGTTCTTACCTCTTTCCCCTTTTTCTTCTTTCCTCGA  840
OariSLCO2A1  CTGCTCATTTCCTCGGCCTGCTTGGTTGTCACCTCTTTCCCCTTCTTTTTCTTTCCTCGA  840
SscrSLCO2A1  CTGCTCATTTCCTCGGCCTCCTTGGTTGTCTCCTCTTTCCCCTTTTTTTTCTTTCCTCGA  840
              L  L  I  S  S  A  S  L  V  V  S  S  F  P  F  F  F  F  P  R
                                                                   TD6><ID4
```

```
                            EX6><EX7
HsapSLCO2A1  GCAATGCCCATAGGAGCAAAGAGGGCTCCTGCCACAGCAGATGAAGCAAGGAAGTTGGAG  900
MmusSLCO2A1  GCGATGTCCAGAGGAGCAGAGAGGTC---TGTTATCGCAGAGGAGACCATGAAGATGGAG  897
RnorSLCO2A1  GCAATGTCCAGAGGAGCAGAGAGGTC---TGTTACCGCAGAGGAAACAATGCAGACGGAG  897
BtauSLCO2A1  GCAATACCCACAAAAATGGAGAGGACTCATCCCATGGTGGATGAAGCAAGGAATACGGAA  900
CfamSLCO2A1  GCAATGCTCAGGGAAGCAGAGAGGTCTCCTGCCATAGTGGATGAAGCAAGGAAGATGGAT  900
OariSLCO2A1  GCAATACCCACAGGAACAGAGAGGACTCATCCCATGATGGATGAAGCAAGGAAGATGGAA  900
SscrSLCO2A1  GCGATGTCCAGAGGGATGGAGAGGACCCCTGCCATGGCGGACGAGGCAAGGAAGATGGAG  900
              A  M  S  R  G  M  E  R  T  P  A  M  A  D  E  A  R  K  M  E

                                                    EX7><EX8
HsapSLCO2A1  GAGGCCAAGTCAAGAGGCTCCCTGGTGGATTTCATTAAACGGTTTCCATGCATCTTTCTG  960
MmusSLCO2A1  GAAGACAAGTCAAGAGGCTCCCTGATGGATTTCATTAAACGGTTCCCCCGCATCTTCCTG  957
RnorSLCO2A1  GAGGACAAGTCAAGAGGCTCCCTGATGGATTTCATTAAACGGTTCCCCCGCATCTTCCTG  957
BtauSLCO2A1  GAGGTCAAGTCAAGAAGATCCCTGGTGGATTTCATTAAAAGGTTCCCCCGCATCTTCCTG  960
CfamSLCO2A1  GAGGCCAAGCCAAGAAGCTCCCTGGTGGATTTCATTAAACGGTTTCCTCGCATCTTCCTG  960
OariSLCO2A1  GAGGCCAAGTCAAG---ATCCCTGGTGGATTTCATTAAAAGGTTCCCCCGCATCTTCCTG  957
SscrSLCO2A1  GAGGCCAAGCCAAGGAGCTCCCTGGTGGATTTCATTAAACGGTTCCCCCGCATCTTCCTG  960
              E  A  K  P  R  S  S  L  V  D  F  I  K  R  F  P  R  I  F  L

HsapSLCO2A1  AGGCTCCTGATGAACTCACTCTTCGTCCTGGTGGTCCTGGCCCAGTGCACCTTCTCCTCC  1020
MmusSLCO2A1  AGGCTGCTGATGAACCCACTCTTCATGCTGGTGGTCCTGAGCCAGTGCACCTTCTCCTCA  1017
RnorSLCO2A1  AGGCTGCTGATGAACCCGCTCTTCATGCTGGTGGTCCTGAGCCAGTGTACCTTCTCCTCA  1017
BtauSLCO2A1  AGGCTCCTGATGAACCCACTCTTCATGCTGGTGGTCCTGGCTCAGTGCACCTTCTCCTCC  1020
CfamSLCO2A1  AAGCTGCTGATGAACCCACTCTTCATGCTGGTGGTCCTGGCCCAGTGCACCTTCTCCTCG  1020
OariSLCO2A1  AGGCTCCTGATGAACTCACTCTTCGTCCTGGTGGTCCTGGCCCAGTGCACCTTTTCCTCC  1017
SscrSLCO2A1  CGGCTCCTGATGAACCCGCTCTTCATGCTGGTGGTCCTGGCCCAGTGCACCTTCTCCTCT  1020
              R  L  L  M  N  P  L  F  M  L  V  V  L  A  Q  C  T  F  S  S
           ID4><TD7

HsapSLCO2A1  GTCATTGCTGGCCTCTCCACCTTCCTCAACAAGTTCCTGGAGAAGCAGTATGGCACCTCA  1080
MmusSLCO2A1  GTCATTGCTGGCCTCTCCACGTTCCTCAACAAGTTCCTGGAGAAGCAGTACGATGCCTCG  1077
RnorSLCO2A1  GTCATCGCTGGCCTCTCCACGTTCCTCAACAAGTTCCTGGAGAAGCAGTATGGAGCCACG  1077
BtauSLCO2A1  GTCATCGCTGGCCTCTCTACCTTCCTCAACAAGTTCCTGGAGAAGCAGTACGGCGCCTCC  1080
CfamSLCO2A1  GTCATTGCTGGCCTCTCCACATTCCTCAACAAGTTTTTGGAGAAGCAGTATGGTGCCTCC  1080
OariSLCO2A1  GTCATTGCCGGCCTCTCTACCTTCCTCAACAAGTTCCTGGAGAAGCAGTACGGCGCCTCC  1077
SscrSLCO2A1  GTCATTGCCGGCCTCTCCACCTTCCTCAACAAGTTCCTCGAGAAGCAGTATGGCGCCTCG  1080
              V  I  A  G  L  S  T  F  L  N  K  F  L  E  K  Q  Y  G  A  S
                   TD7><ED4

                          EX8><EX9
HsapSLCO2A1  GCAGCCTATGCCAACTTCCTCATTGGTGCTGTGAACCTCCCTGCTGCAGCCTTGGGGATG  1140
MmusSLCO2A1  GCAGCCTATGCCAACTTACTCATTGGTGCTGTGAACCTTCCAGCTGCGGCCTTGGGGATG  1137
RnorSLCO2A1  GCAGCCTATGCCAACTTCCTCATCGGTGCTGTAAATCTTCCGGCTGCAGCCTTGGGGATG  1137
BtauSLCO2A1  GCAGCTTATGCCAACTTCCTCATCGGTGCTGTGAACCTCCCTGCTGCGGCCTTGGGGATG  1140
CfamSLCO2A1  GCAGCCTATGCCAACTTCCTCATCGGTGCTGTGAACCTCCCAGCTGCAGCACTAGGAATG  1140
OariSLCO2A1  GCAGCCTATGCCAACTTCCTCATCGGTGCTGTGAACCTCCCTGCTGCGGCCTTAGGGATG  1137
SscrSLCO2A1  GCGGCCTATGCCAACTTCCTCATCGGTGCTGTGAACCTCCCGGCCGCGGCCTTGGGAATG  1140
              A  A  Y  A  N  F  L  I  G  A  V  N  L  P  A  A  A  L  G  M
             ED4><TD8
```

```
HsapSLCO2A1  CTGTTTGGAGGAATCCTCATGAAGCGCTTTGTTTTCTCTCTACAAACCATTCCCCGCATA  1200
MmusSLCO2A1  CTGTTTGGAGGAATCCTCATGAAGCGTTTTGTTTTCCCTCTGCAAACCATCCCCCGAGTA  1197
RnorSLCO2A1  CTGTTTGGAGGAATCCTCATGAAGCGTTTTGTTTTCCCTCTGCAAACTATCCCCCGAGTG  1197
BtauSLCO2A1  CTGCTTGGAGGGATCCTCATGAAGCGCTGTGCTTTCTCTCTGCAAACCATTCCCCGTGTA  1200
CfamSLCO2A1  CTGTTCGGAGGAATCCTCATGAAGCGTTTTGTTTTCTCTCTGCAAACCATTCCCCGCGTG  1200
OariSLCO2A1  CTGCTTGGAGGGATCCTCATGAAGCGCTGTGTTTTCTCTCTGCAAACCATTCCCCGTGTA  1197
SscrSLCO2A1  CTGTTTGGAGGAATCCTCATGAAGCGTTTCGTTTTCTCCCTGCAAACCATTCCCCGAGTG  1200
              L  F  G  G  I  L  M  K  R  F  V  F  S  L  Q  T  I  P  R  V
                     TD8><ID5                                  ID5><TD9

HsapSLCO2A1  GCTACCACCATCATCACCATCTCCATGATCCTTTGTGTTCCTTTGTTCTTCATGGGATGC  1260
MmusSLCO2A1  GCTGCCACCATCATGACCATCTCCATAATCCTATGTGCCCCTCTCTTCTTTATGGGATGT  1257
RnorSLCO2A1  GCTGCCACCATCATCACCATCTCCATGATCCTCTGTGTACCTCTCTTCTTTATGGGATGC  1257
BtauSLCO2A1  GCTGCCACCATCATCATCATCTCCATGATCCTTTGTGCCCCTCTGTTCTTCATGGGCTGC  1260
CfamSLCO2A1  GCTGCCACCATCATCACCATCTCCATGATTCTCTGCGCCCCTCTCTTCTTCATGGGATGC  1260
OariSLCO2A1  GCCGCCACCATCATCATCGTCTCCATGGTGCTCTGTGCCCCTCTGTTCTTCATGGGCTGC  1257
SscrSLCO2A1  GCTGCCACCATCATCACCATCTCCATGATCCTCTGTGCCCCTCTGTTCTTCATGGGCTGC  1260
              A  A  T  I  I  T  I  S  M  I  L  C  A  P  L  F  F  M  G  C
                                                               TD9><ED5

                                             EX9><EX10
HsapSLCO2A1  TCCACCCCAACTGTGGCCGAAGTCTACCCCCCTAGCACATCAAGTTCTATACATCCGCAG  1320
MmusSLCO2A1  TCCACACCCGCCGTGGCTGAGGTCTACCCTCCCAGCACACCAAGTTCTATACATCCGCAG  1317
RnorSLCO2A1  TCCACATCAGCCGTGGCTGAGGTCTACCCTCCCAGCACATCAAGTTCTATACATCCGCAG  1317
BtauSLCO2A1  TCCACCCCGCTCGTGGCGGAGGTCTACCCCCCGAGCACATCAAGTTCTATACGTCCACAA  1320
CfamSLCO2A1  TCCACCCCCACGGTAGCGGAGGTCTATCCCCCCAGGACATCAAGTCCTATACATCCGCAG  1320
OariSLCO2A1  TCCACCCCGCTCGTGTCGGAGGTCTACCCCCCAAGCACATCAAGTTCTATACGTCCACAG  1317
SscrSLCO2A1  TCCACTCCCAAGGTGGCTGAGGTCTACCCTCCCAGCACATCAAGTTCTATACATCCACAG  1320
              S  T  P  K  V  A  E  V  Y  P  P  S  T  S  S  S  I  H  P  Q

HsapSLCO2A1  T---CTCCTGCCTGCCGCAGGGACTGCTCGTGCCCAGATTCTATCTTCCACCCGGTCTGT  1377
MmusSLCO2A1  C---CTCCCGCCTGCCGCAGGGACTGCCTGTGCCCAGATTCCGTCTTCCACCCTGTCTGC  1374
RnorSLCO2A1  CAGCCTCCTGCCTGCCGCAGGGACTGCTCGTGCCCAGATTCCTTCTTCCACCCAGTCTGT  1377
BtauSLCO2A1  C---CTCTCCCCTGCCGCCAGGGCTGCTCGTGCCCAGATTCTGTCTTCCACCCGGTCTGT  1377
CfamSLCO2A1  C---CTCCTGCCTGCCGCAGGCACTGCTCGTGCCCAGACTCCATCTTCCACCCAGTGTGT  1377
OariSLCO2A1  C---CTCCCGCCTGCCGCCAGGACTGCTCGTGCCCAGATTCTGTCTTCCACCCTGTCTGT  1374
SscrSLCO2A1  C---CTCTCGCCTGCCGCAAGGACTGCTCGTGCCCAGATTCCCTCTTCCACCCGGTTTGC  1377
                  P  L  A  C  R  K  D  C  S  C  P  D  S  L  F  H  P  V  C

HsapSLCO2A1  GGAGACAATGGAATCGAGTACCTCTCCCCTTGCCATGCCGGCTGCAGCAACATCAACATG  1437
MmusSLCO2A1  GGAGACAATGGAGTCGAGTACCTCTCCCCCTGCCATGCTGGCTGCAGCAGCCTCAACGTG  1434
RnorSLCO2A1  GGAGACAATGGAGTCGAGTACGTTTCCCCTTGCCACGCCGGCTGCAGCAGCACCAACACA  1437
BtauSLCO2A1  GGAGACGATGGGATTGAATACCTCTCCCCGTGCCACGCTGGCTGCAGTGAAGTCAACTTC  1437
CfamSLCO2A1  GGAGACAACGGAGTAGAGTACCTCTCGCCGTGCCACGCAGGCTGCAGCGAAATCAACGTC  1437
OariSLCO2A1  GGAGACAATGGGGTGGAATACCTCTCCCCTTGCCACGCTGGCTGCAGTGAAGTCAACTTC  1434
SscrSLCO2A1  GGAGACAATGGGGTTGAGTACCTCTCCCCCTGCCACGCCGGCTGCAGTGACATCAATGTC  1437
              G  D  N  G  V  E  Y  L  S  P  C  H  A  G  C  S  D  I  N  V
```

```
                       EX10><EX11
HsapSLCO2A1  AGCTCTGCAACCTCCAAGCAACTGATCTATTTGAACTGCAGCTGTGTGACCGGGGGATCC 1497
MmusSLCO2A1  AGCTCAGCAGCTTCTAAGCAACCAATCTATTTGAACTGTAGCTGTGTGACTGGAGGATCC 1494
RnorSLCO2A1  AGCTCAGAAGCTTCTAAGGAACCGATCTACTTGAACTGCAGCTGTGTGAGTGGAGGATCG 1497
BtauSLCO2A1  AGCTCCATAGCTCTCAAGCAACCGATCTACTTGAACTGCAGCTGCGTTAACAGGGGATCT 1497
CfamSLCO2A1  ACCTCCATAGCCTCCAAGCAGCTGATGTATCTGAACTGCAGCTGTGTGACCGGGGCATCT 1497
OariSLCO2A1  AGCTCTATAGCTCTCAAGAAACCGATCTACTTGAACTGCAGCTGCGTCAGCGGGGGATCT 1494
SscrSLCO2A1  AGCTCTATAGCCTCTAAGCAACTGACCTACCTGAACTGCAGCTGTGTGAGTGGGGCATCA 1497
              S  S  I  A  S  K  Q  L  T  Y  L  N  C  S  C  V  S  G  A  S

HsapSLCO2A1  GCTTCAGCAAAGACAGGATCGTGCCCTGTCCCCTGTGCCCACTTCCTGCTCCCGGCCATC 1557
MmusSLCO2A1  GCATCGGCCAAGACAGGCTCCTGCCCTACATCCTGTGCACAGCTGCTGCTCCCGTCCATC 1554
RnorSLCO2A1  GCGTCA--CAAGACAGGCTCATGCCCCACGTCCTGCGCGCA-CTACTGCTCCCGTCCATC 1554
BtauSLCO2A1  GGTTCAGCGAAGACAGGCCCGTGTCCCGTCTCCTGCGCCCACTTCCTGCTCCCAACCATC 1557
CfamSLCO2A1  GCTTCAGCCAAGACGGGATCGTGCCCCATCCCCTGTGCCCACTTCCTGCTCCCAGCCATC 1557
OariSLCO2A1  GCTTCAGCGAAGACAGGCCCGTGTCCCGTCTCCTGCGCCCACTTCCTGCTCCCGACCACC 1554
SscrSLCO2A1  GCTTCAGCAAAGACGGGCCCGTGTCCCATCCCCTGCGCCCACTTTCTGCTCCCAGCCATC 1557
              A  S  A  K  T  G  P  C  P  I  P  C  A  H  F  L  L  P  A  I
                                             ED5><TD10

HsapSLCO2A1  TTCCTCATCTCCTTCGTGTCCCTGATAGCCTGCAT----CTCCCACAACCCCCTCTACAT 1613
MmusSLCO2A1  TTCCTCATCTCCTTTGTGGCACTCATTGCCTGCGT----CTCCCACAACCCCCTCTACAT 1610
RnorSLCO2A1  TTCCTCATTTCCTTTGCGGCGCTCATTGCCTGCAT----CTCCCACAACCCGCTCTACAT 1610
BtauSLCO2A1  TTCCTCATCTCCTTTGCGGCGCTCATAGCCTGCAT----CTCGCACAACCCCCTCTACAT 1613
CfamSLCO2A1  TTCCTCATCTCCTTCGTGGCCCTCATAGCCTGCAT----CTCACACAACCCACTCTACAT 1613
OariSLCO2A1  TTCCTCATCTCCTTCGTGGCGCTCATAGCCTGCGTATCTCTCGCACAACCCC-TCTACAT 1613
SscrSLCO2A1  TTCCTGATCTCCTTCGTGGCCCTCATAGCCTGCGT----CTCCCACAACCCACTCTACAT 1613
              F  L  I  S  F  V  A  L  I  A  C  V     S  H  N  P  L  Y  M
                                                            TD10><ID6

                   EX11><EX12
HsapSLCO2A1  GATGGTTCTGCGTGTGGTGAACCAGGAGGAAAAGTCATTTGCCATCGGGGTGCAGTTCTT 1673
MmusSLCO2A1  GATGGTCCTACGTGTGGTGAACCAGGATGAAAAGTCGTTTGCCATTGGAGTACAGTTCTT 1670
RnorSLCO2A1  GATGGTCCTTCGCGTGGTGAACCAGGATGAAAAGTCGTTTGCCATTGGGGTACAGTTCTT 1670
BtauSLCO2A1  GATGGTGCTGCGTGTGGTGAACCAGGATGAAAAGTCCTTCGCCATCGGAGTGCAGTTCTT 1673
CfamSLCO2A1  GATGGTTCTGCGTGTGGTGAACCAGGAAGAAAAGTCATTTGCCATTGGGGTACAGTTCTT 1673
OariSLCO2A1  GATGGTGCTGCGTGTGGTGAACCGGAGGAAAAAGTCCTTCGCCATCGGAGTACAGTTCTT 1673
SscrSLCO2A1  GATGGTACTGCGTGTGGTGAACCAGGAGGAAAAGTCATTTGCCATCGGGGTACAGTTCTT 1673
              M  V  L  R  V  V  N  Q  E  E  K  S  F  A  I  G  V  Q  F  L
                                                   ID6><TD11

                      EX12><EX13
HsapSLCO2A1  GTTGATGCGCTTGCTGGCCTGGCTGCCATCTCCAGCCCTCTATGGCCTCACCATTGACCA 1733
MmusSLCO2A1  GTTGATGCGTTTGCTGGCCTGGCTGCCATCTCCATCCCTTTATGGCCTGCTCATCGACTC 1730
RnorSLCO2A1  GTTGATGCGCTTGCTGGCCTGGCTGCCGCTCCATCCCTTTATGGCCTCCTCATCGACTC 1730
BtauSLCO2A1  GCTGATGCGTCTGCTGGCCTGGTTGCCGTCTCCAGCCCTCTATGGCCTCACCATTGACTA 1733
CfamSLCO2A1  GCTGATGCGCCTGCTGGCCTGGCTGCCGTCTCCAGCCCTCTACGGCCTCACCATCGACTA 1733
OariSLCO2A1  GCTGATGCGTCTGCTGGCTTGGCTGCCGGCTCCAGCTCTCTATGGCCTCACCATTGACTA 1733
SscrSLCO2A1  GCTGATGCGTCTGCTGGCCTGGCTGCCATCTCCAGCCCTCTACGGCCTCACCATCGACTA 1733
              L  M  R  L  L  A  W  L  P  S  P  A  L  Y  G  L  T  I  D  Y
                                                            TD11><ED6
```

```
HsapSLCO2A1  CTCCTGCATCCGGTGGAACTCGCTGTGCTTGGGGAGGCGAGGGGCCTGCGCCTACTATGA  1793
MmusSLCO2A1  CTCCTGTATCCGGTGGAACTACCTATGCTCAGGGAGACGAGGGGCCTGTGCGTATTATGA  1790
RnorSLCO2A1  CTCCTGTGTCCGGTGGAACTACCTATGCTCAGGGAGACGAGGGGCCTGTGCGTATTATGA  1790
BtauSLCO2A1  CTCCTGCATCCTGTGGAGCGCAAAGTGCTCGGGAAGGAGAGGGGCCTGTGTCTACTATGA  1793
CfamSLCO2A1  CTCCTGCATCAAGTGGAACTTTCAGTGCTCAGGGAGGCGCGGGGCCTGTGCCTACTATGA  1793
OariSLCO2A1  CTCCTGTATCCTGTGGAGCACGAAGTGCTCGGGGAGGAGAGGGGCCTGTGCCTACTACGA  1793
SscrSLCO2A1  CTCCTGCATCCGGTGGAGCTCCCAGTGCTCGGGGAGGCGGGGGGCCTGTGCTTACTACGA  1793
               S  C  I  R  W  S  S  Q  C  S  G  R  R  G  A  C  A  Y  Y  D

                                 EX13><EX14
HsapSLCO2A1  CAACGATGCTCTCCGAGACAGGTACCTGGCCTGCAGATGGGCTACAAGGCGCTGGCAT  1853
MmusSLCO2A1  CAACGATGCTCTTCGAAACAGGTACCTGGCCTACAGGTAATCTACAAGGTCTTGGCAC  1850
RnorSLCO2A1  CAACGATGCTCTCCGAAACAGGTACCTGGCCTACAGATGGTCTACAAGGCCTTGGCAC  1850
BtauSLCO2A1  CAACAATGCTCTCCGGAACAGGTACCTGGCCTGCAGGTGGCCTACAAGGCTCTGGCTC  1853
CfamSLCO2A1  CAATGATGCTCTCCGAGACAGGTACCTGGCCTGCAGGTGGGCTACAAGGCGCTGGTGC  1853
OariSLCO2A1  CAACAACGCTCTCCGGAACAGGTATCTGGCCTGCAGGTGGCCTACAAGGCCCTGGCGC  1853
SscrSLCO2A1  CAATGACGCTCTCCGAGACAGGTACCTGGCCTGCAGGTGGGCTACAAGGCTCTGGCAC  1853
               N  D  A  L  R  D  R  Y  L  G  L  Q  V  G  Y  K  A  L  G  T
                                   ED6><TD12

HsapSLCO2A1  GCTGCTGCTTTGCTTCATCAGCTGGAGGGTGAAGAAGAACAAGGAGTACAACGTGCAG--  1911
MmusSLCO2A1  ACTGCTGCTCTTCTTCATCAGCTGGAGGGTGAAGAAGAACAGGGAATACAGTCTGCAGGA  1910
RnorSLCO2A1  ACTGCTGCTCTTCTTCATCAGCTGGAGGATGAAGAAGAACAGGGAATACAGCCTGCAGGA  1910
BtauSLCO2A1  AGTGCTGCTTATCTTCATCAGCTGGCGGGTGAAGAAGAACAAAGAGTACAATGTTCAGGA  1913
CfamSLCO2A1  GCTGCTGCTCCTATTCATCAGCTGGCGGGTGAAGAAGAGCAAGGAGTACAATGTGCAGGA  1913
OariSLCO2A1  GGTGCTGCTTATCTTCATCAGCTGGCGGGTGAAGAAGAACAAGGAGTACAACGTGCAGGA  1913
SscrSLCO2A1  GCTGCTGCTCCTCTTCACCAGCTGGCGGGTGAAGAAGAACAAGGAATACAATGTGCAGGA  1913
               L  L  L  L  F  T  S  W  R  V  K  K  N  K  E  Y  N  V  Q  E
                                     TM12><ID7

HsapSLCO2A1  -AAGGCGGCAGGCCTCATCTGA  1932
MmusSLCO2A1  GAATGCCTCGGGCCTCATCTGA  1932
RnorSLCO2A1  GAACACCTCAGGCCTCATCTGA  1932
BtauSLCO2A1  GAAGGCGGCCGGCCTCATCTGA  1935
CfamSLCO2A1  GAAGGCCGCAGGCCTCATCTGA  1935
OariSLCO2A1  GAAGGCTGCCAGCCTCATCTGA  1935
SscrSLCO2A1  GAAGGCCGCCGGCCTCATCTGA  1935
               K  A  A  G  L  I  *
```
