# Supplementary material for: Molecular cloning and characterization of the porcine prostaglandin transporter (SLCO2A1): evaluation of its role in F4 mediated neonatal diarrhoea
Source: BMC Genet. 2009 Oct 6;10:64. doi: 10.1186/1471-2156-10-64 (PMC2763009; doi:10.1186/1471-2156-10-64)
Supplement: Additional file 2 — Amino acid sequence alignment between porcine SLCO2A1 and its published orthologs in man, mouse, rat, cow, dog and sheep. Comparative SLCO2A1 amino acid sequence alignment with indication of the conserved sequences and the predicted protein domains. [file 1471-2156-10-64-S2.PDF]

Additional file 2. Amino acid sequence alignment between porcine (*Sscr*) SLCO2A1 [GenBank:NM_001123195] and its published orthologs in man (*Hsap*) [Genbank:NM_005630], mouse (*Mmus*) [Genbank:NM_033314], rat (*Rnor*) [Genbank:NM_022667], cow (*Btau*) [Genbank:NM_174829], dog (*Cfam*) [Genbank:NM_001011558] and sheep (*Oari*) [Genbank:DQ026455]. Identical amino acids are in black, similar ones in grey. Predicted intracellular (ID), transmembrane (TD) and extracellular (ED) domains are indicated.

```
            |--------------ID1------------||---------TD1-------||----ED1
HsapSLCO2A1 MGLLPKLGVSQGSDTSTSRAGRCARSVFGNIKVFVLCQGLLQLCQLLYSAYFKSSLTTIE 60
MmusSLCO2A1 MGLLPKPGARQGSGTSSVPARRCSRSVFNNIKVFVLCHGLLQLCQLLYSAYFKSSLTTIE 60
RnorSLCO2A1 MGLLLKPGARQGSGTSSVPDRRCPRSVFSNIKVFVLCHGLLQLCQLLYSAYFKSSLTTIE 60
BtauSLCO2A1 MGFLPKPGARQGSAASTGQAGCSPRSIFSNIKVFVLCHGLLQLCQLLYSAYFRSSLTTIE 60
CfamSLCO2A1 MGLLPKPDARRGSDASPGRPRRCPRSVFSNIKVFVLCHGLLQLCQLLHSAYFKSSLTTIE 60
OariSLCO2A1 MGLLPKLGARQGSAASAGRAGCNPRSIFSNIKVFVLCHGLLQLCQLLYSAYFKSSLTTIE 60
SscrSLCO2A1 MGLLPEPSARQSSGASAGRASRCPRSIFSNIKVFVLCHGLLQLCQLLYSAYFKSSLTTIE 60

            -------||---------TD2----------||-ID2-||----------TD3-------
HsapSLCO2A1 KRFGLSSSSSGLISSLNEISNAILIIFVSYFGSRVHRPRLIGIGGLFLAAGAFILTLPHF 120
MmusSLCO2A1 KRFGLSSSSSGLISSLNEISNAILIIFVSYFGSRVNRPRMIGIGGLLLAAGAFVLTLPHF 120
RnorSLCO2A1 KRFGLSSSSSGLISSLNEISNATLIIFISYFGSRVNRPRMIGIGGLLLAAGAFVLTLPHF 120
BtauSLCO2A1 KRFGLSSSSSGFISSLNEISNAVLIIFVSYFGSRVHRPRLIGIGGLLLALGAFILTLPHF 120
CfamSLCO2A1 KRFGLSSSSSGLISSLNEISNAILIIFVSYFGSRVHRPRLIGIGGLLLASGAFILTLPHF 120
OariSLCO2A1 KRFGLSSSSSGFISSLNEISNAILIIFVSYFGSRVHRPRLIGIGGLLLALGAFVLTLPHF 120
SscrSLCO2A1 KRFGLSSSSSGLISSLNEISNAVLIIFVSYFGSRVHRPRLIGIGGLLLASGAFVLTLPHF 120

            -||---------------------ED2------------------||--------TD4
HsapSLCO2A1 LSEPYQYTLASTGNNSRLQAELCQKHWQDLPPSKCHSTTQNPQKETSSMWGLMVVAQLLA 180
MmusSLCO2A1 LSEPYQYASTTAGNSSHFQTDLCQKHLPGLLPSKCHSTVPDTQKETSSMWSLMVVAQLLA 180
RnorSLCO2A1 LSEPYQYTSTTDGNRSSFQTDLCQKHFGALPPSKCHSTVPDTHKETSSLWGLMVVAQLLA 180
BtauSLCO2A1 LSEPYQYTKTIMGNSSHLQTELCQKSWQGLPPSKCHSSPQDSQKETSSMWGLMVIAQLLA 180
CfamSLCO2A1 LSEPYQYTLASVGNSSHFQAELCQKHWQDLPPSKCHSTTQDSRKETSSMWGLMVVAQLLA 180
OariSLCO2A1 LSEPYQYTKTIMGNSSHLQTELCQKAWQGLPPSKCHSSPQDSRKETSSMWGLMVIAQLLA 180
SscrSLCO2A1 LSEPYQYTVTIVGNSSRLQAELCQKHWQGLPPSKCHSSIQDSRKETSSVWGLMVVAQLLA 180

            -----------||----ID3----||---------TD5----------||-------ED3
HsapSLCO2A1 GIGTVPIQPFGISYVDDFSEPSNSPLYISILFAISVFGPAFGYLLGSIMLQIFVDYGRVN 240
MmusSLCO2A1 GVGTVPIQPFGISYVDDFAEPTNSPLYISILFAIAVFGPAFGYLLGSVMLRIFVDYGRVD 240
RnorSLCO2A1 GIGTVPIQPFGISYVDDFAEPTNSPLYISILFAIAVFGPAFGYLLGSVMLRIFVDYGRVD 240
BtauSLCO2A1 GIGTVPIQPFGISYVDDFSEPNNSPLYISILFAIAVFGPAFGYLLGSVMLQIFVDYGRVD 240
CfamSLCO2A1 GVGTVPIQPFGISYVDDFSEPNNSPLYISILFAISVFGPAFGYLLGSVMLQIFVDYGRVD 240
OariSLCO2A1 GIGTVPIQPFGISYVDDFSEPNNSPLYISILFAIAVFGPAFGYLLGSVMLQIFVDYGRVD 240
SscrSLCO2A1 GIGTVPIQPFGISYVDDFSEPNNSPLYISILFAISVFGPAFGYLLGSVMLQIFVDYGRVD 240

            --------------||---------TD6----------||-----------------ID4
HsapSLCO2A1 TAAVNLVPGDPRWIGAWWLGLLISSALLVLTSFPFFFFPRAMPIGAKRAPATADEARKLE 300
MmusSLCO2A1 TATVNLSPGDPRWIGAWWLGLLISSGFLIVTSLPFFFFPRAMSRGAERS-VIAEETMKME 299
RnorSLCO2A1 TATVNLSPGDPRWIGAWWLGLLISSGFLIVTSLPFFFFPRAMSRGAERS-VTAEETMQTE 299
BtauSLCO2A1 TASVNLSPGDPRWIGAWWLGLLISSACLVVTSFPFFFFPRAIPTKMERTHPMVDEARNTE 300
CfamSLCO2A1 TAAVNLSPGDPRWIGAWWLGLLISSASLVLTSFPFFFFPRAMLREAERSPAIVDEARKMD 300
OariSLCO2A1 TASVNLSPGDPRWIGAWWLGLLISSACLVVTSFPFFFFPRAIPTGTERTHPMMDEARKME 300
SscrSLCO2A1 TATVNLSPGDPRWIGAWWLGLLISSASLVVSSFPFFFFPRAMSRGMERTPAMADEARKME 300
```

```
                 ---------------------||---------TD7----------||--------ED4---
HsapSLCO2A1      EAKSRGSLVDFIKRFPCIFLRLLMNSLFVLVVLAQCTFSSVIAGLSTFLNKFLEKQYGTS  360
MmusSLCO2A1      EDKSRGSLMDFIKRFPRIFLRLLMNPLFMLVVLSQCTFSSVIAGLSTFLNKFLEKQYDAS  359
RnorSLCO2A1      EDKSRGSLMDFIKRFPRIFLRLLMNPLFMLVVLSQCTFSSVIAGLSTFLNKFLEKQYGAT  359
BtauSLCO2A1      EVKSRRSLVDFIKRFPRIFLRLLMNPLFMLVVLAQCTFSSVIAGLSTFLNKFLEKQYGAS  360
CfamSLCO2A1      EAKPRSSLVDFIKRFPRIFLKLLMNPLFMLVVLAQCTFSSVIAGLSTFLNKFLEKQYGAS  360
OariSLCO2A1      EAKSR-SLVDFIKRFPRIFLRLLMNSLFVLVVLAQCTFSSVIAGLSTFLNKFLEKQYGAS  359
SscrSLCO2A1      EAKPRSSLVDFIKRFPRIFLRLLMNPLFMLVVLAQCTFSSVIAGLSTFLNKFLEKQYGAS  360

                 -||----------TD8---------||----ID5----||--------TD9-------||
HsapSLCO2A1      AAYANFLIGAVNLPAAALGMLFGGILMKRFVFSLQTIPRIATTIITISMILCVPLFFMGC  420
MmusSLCO2A1      AAYANLLIGAVNLPAAALGMLFGGILMKRFVFPLQTIPRVAATIMTISIILCAPLFFMGC  419
RnorSLCO2A1      AAYANFLIGAVNLPAAALGMLFGGILMKRFVFPLQTIPRVAATIITISMILCVPLFFMGC  419
BtauSLCO2A1      AAYANFLIGAVNLPAAALGMLLGGILMKRCAFSLQTIPRVAATIIIISMILCAPLFFMGC  420
CfamSLCO2A1      AAYANFLIGAVNLPAAALGMLFGGILMKRFVFSLQTIPRVAATIITISMILCAPLFFMGC  420
OariSLCO2A1      AAYANFLIGAVNLPAAALGMLLGGILMKRCVFSLQTIPRVAATIIIVSMVLCAPLFFMGC  419
SscrSLCO2A1      AAYANFLIGAVNLPAAALGMLFGGILMKRFVFSLQTIPRVAATIITISMILCAPLFFMGC  420

                 ------------------------------------ED5---------------------
HsapSLCO2A1      STPTVAEVYPPSTSSSIHPQ-SPACRRDCSCPDSIFHPVCGDNGIEYLSPCHAGCSNINM  479
MmusSLCO2A1      STPAVAEVYPPSTPSSIHPQ-PPACRRDCLCPDSVFHPVCGDNGVEYLSPCHAGCSSLNV  478
RnorSLCO2A1      STSAVAEVYPPSTSSSIHPQQPPACRRDCSCPDSFFHPVCGDNGVEYVSPCHAGCSSTNT  479
BtauSLCO2A1      STPLVAEVYPPSTSSSIRPQ-PLPCRQGCSCPDSVFHPVCGDDGIEYLSPCHAGCSEVNF  479
CfamSLCO2A1      STPTVAEVYPPRTSSPIHPQ-PPACRRHCSCPDSIFHPVCGDNGVEYLSPCHAGCSEINV  479
OariSLCO2A1      STPLVSEVYPPSTSSSIRPQ-PPACRQDCSCPDSVFHPVCGDNGVEYLSPCHAGCSEVNF  478
SscrSLCO2A1      STPKVAEVYPPSTSSSIHPQ-PLACRKDCSCPDSLFHPVCGDNGVEYLSPCHAGCSDINV  479

                 --------------------------------||----------TD10---------||-
HsapSLCO2A1      SSATSKQLIYLNCSCVTGGSASAKTGSCPVPCAHFLLPAIFLISFVSLIACISHN-PLYM  538
MmusSLCO2A1      SSAASKQPIYLNCSCVTGGSASAKTGSCPTSCAQLLLPSIFLISFVALIACVSHN-PLYM  537
RnorSLCO2A1      SSEASKEPIYLNCSCVSGGSASQDR-LMPHVLRALLLPSIFLISFAALIACISHN-PLYM  537
BtauSLCO2A1      SSIALKQPIYLNCSCVNRGSGSAKTGPCPVSCAHFLLPTIFLISFAALIACISHN-PLYM  538
CfamSLCO2A1      TSIASKQLMYLNCSCVTGASASAKTGSCPIPCAHFLLPAIFLISFVALIACISHN-PLYM  538
OariSLCO2A1      SSIALKKPIYLNCSCVSGGSASAKTGPCPVSCAHFLLPTTFLISFVALIACVSLAQPLYM  538
SscrSLCO2A1      SSIASKQLTYLNCSCVSGASASAKTGPCPIPCAHFLLPAIFLISFVALIACVSHN-PLYM  538

                 -----ID6-----||---------TD11---------||-----------ED6-------
HsapSLCO2A1      MVLRVVNQEEKSFAIGVQFLLMRLLAWLPSPALYGLTIDHSCIRWNSLCLGRRGACAYYD  598
MmusSLCO2A1      MVLRVVNQDEKSFAIGVQFLLMRLLAWLPSPSLYGLLIDSSCIRWNYLCSGRRGACAYYD  597
RnorSLCO2A1      MVLRVVNQDEKSFAIGVQFLLMRLLAWLPAPSLYGLLIDSSCVRWNYLCSGRRGACAYYD  597
BtauSLCO2A1      MVLRVVNQDEKSFAIGVQFLLMRLLAWLPSPALYGLTIDYSCILWSAKCSGRRGACVYYD  598
CfamSLCO2A1      MVLRVVNQEEKSFAIGVQFLLMRLLAWLPSPALYGLTIDYSCIKWNFQCSGRRGACAYYD  598
OariSLCO2A1      MVLRVVNRRKKSFAIGVQFLLMRLLAWLPAPALYGLTIDYSCILWSTKCSGRRGACAYYD  598
SscrSLCO2A1      MVLRVVNQEEKSFAIGVQFLLMRLLAWLPSPALYGLTIDYSCIRWSSQCSGRRGACAYYD  598

                 ------||--------TD12-------||------ID7-------|
HsapSLCO2A1      NDALRDRYLGLQMGYKALGMLLLCFISWRVKKNKEYNVQK-AAGLI  643
MmusSLCO2A1      NDALRNRYLGLQVIYKVLGTLLLFFISWRVKKNREYSLQENASGLI  643
RnorSLCO2A1      NDALRNRYLGLQMVYKALGTLLLFFISWRMKKNREYSLQENTSGLI  643
BtauSLCO2A1      NNALRNRYLGLQVAYKALGSVLLIFISWRVKKNKEYNVQEKAAGLI  644
CfamSLCO2A1      NDALRDRYLGLQVGYKALGALLLLFISWRVKKSKEYNVQEKAAGLI  644
OariSLCO2A1      NNALRNRYLGLQVAYKALGAVLLIFISWRVKKNKEYNVQEKAASLI  644
SscrSLCO2A1      NDALRDRYLGLQVGYKALGTLLLLFTSWRVKKNKEYNVQEKAAGLI  644
```
