# Supplementary material for: Molecular cloning and characterization of the porcine prostaglandin transporter (SLCO2A1): evaluation of its role in F4 mediated neonatal diarrhoea
Source: BMC Genet. 2009 Oct 6;10:64. doi: 10.1186/1471-2156-10-64 (PMC2763009; doi:10.1186/1471-2156-10-64)
Supplement: Additional file 3 — Porcine SLCO2A1 sequence comparison. Overview in percentage of the porcine SLCO2A1 nucleic acid and amino acid sequence homologies with its published orthologs in man, mouse, rat, cow, dog and sheep. [file 1471-2156-10-64-S3.PDF]

Additional file 3. Porcine *SLCO2A1* sequence comparison [GenBank:NM_001123195].

Species	*Acc. No.*	*Length (bp/aa)*	*bp seq ID*	*aa seq ID*	*aa seq POS*
Man	Genbank:NM_005630	1932 / 643	87%	87%	92%
Mouse	Genbank:NM_033314	1932 / 643	85%	84%	91%
Rat	Genbank:NM_022667	1932 / 643	84%	82%	89%
Cow	Genbank:NM_174829	1935 / 644	88%	87%	92%
Dog	Genbank:NM_001011558	1935 / 644	89%	91%	95%
Sheep	Genbank:DQ026455	1935 / 644	88%	88%	92%
