# Supplementary material for: Molecular cloning and characterization of the porcine prostaglandin transporter (SLCO2A1): evaluation of its role in F4 mediated neonatal diarrhoea
Source: BMC Genet. 2009 Oct 6;10:64. doi: 10.1186/1471-2156-10-64 (PMC2763009; doi:10.1186/1471-2156-10-64)
Supplement: Additional file 4 — SLCO2A1 mRNA expression profiling in the jejunum of 8 pigs with a different F4ab/ac receptor phenotype by RT-PCR. Agarose gels showing 5 overlapping (covering the complete transcript) jejunal SLCO2A1 RT-PCR products of 8 receptor positive/negative pigs. [file 1471-2156-10-64-S4.PDF]

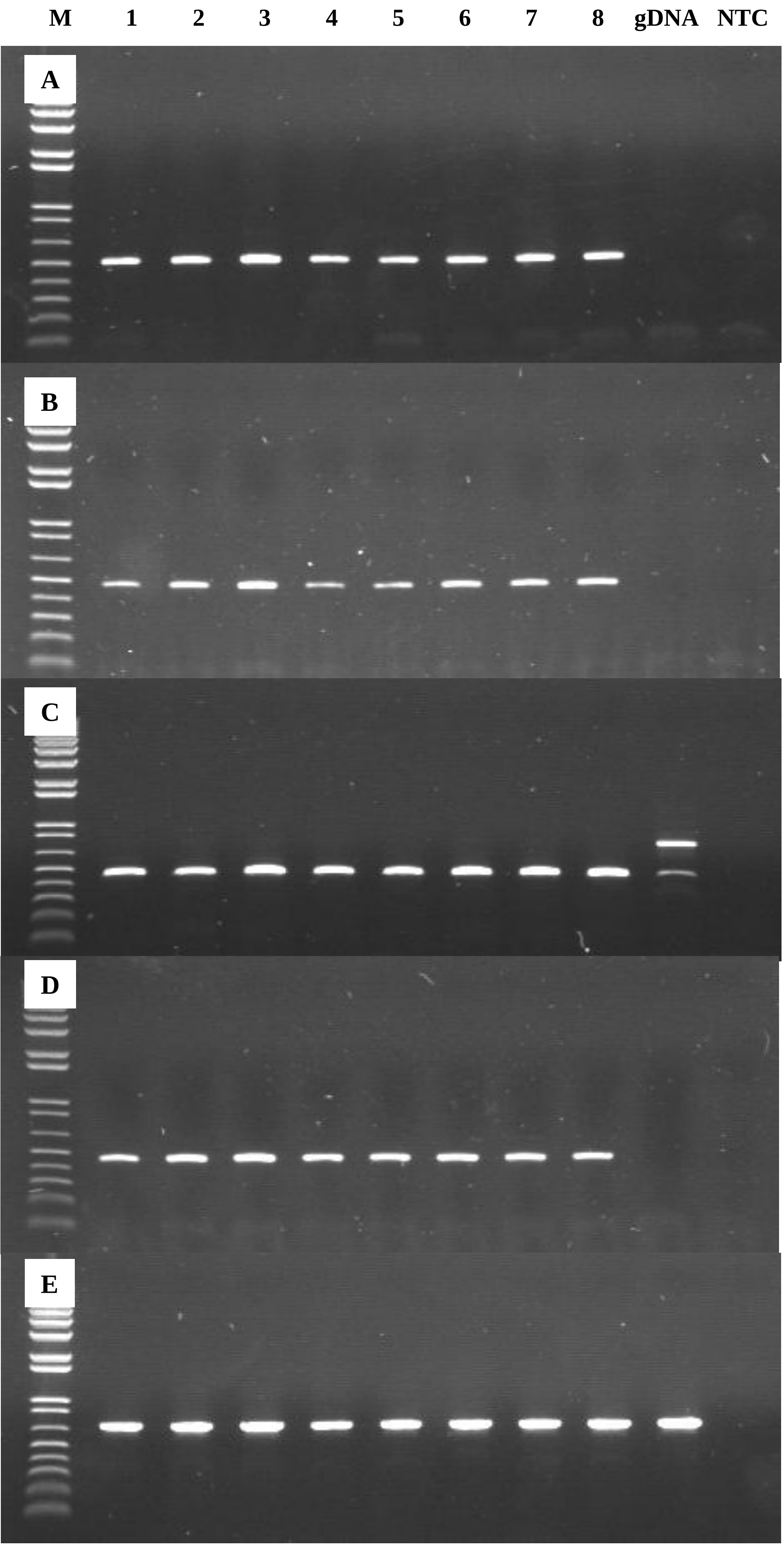

Additional file 4. *SLCO2A1* mRNA expression profiling in the jejunum of 8 pigs (1-8) with a different F4ab/ac receptor phenotype by RT-PCR with different exon spanning primer pairs covering the whole *SLCO2A1* transcript: A) F37/R37, B) F38/R38, C) F18/R18, D) F39/R39 and E) F26/R26 (see additional file 5). M is 1 KB plus DNA ladder (Invitrogen). gDNA is porcine genomic DNA. NTC is no template control.
