# Supplementary material for: Molecular cloning and characterization of the porcine prostaglandin transporter (SLCO2A1): evaluation of its role in F4 mediated neonatal diarrhoea
Source: BMC Genet. 2009 Oct 6;10:64. doi: 10.1186/1471-2156-10-64 (PMC2763009; doi:10.1186/1471-2156-10-64)
Supplement: Additional file 5 — Primer names/sequences/positions, amplicon sizes and PCR conditions. Table including all information needed to perform all PCR/sequencing reactions described in this paper. [file 1471-2156-10-64-S5.PDF]

Additional file 5. Primer names/sequences/positions, amplicon sizes and PCR conditions.

Forward Primer Name<br>Reverse Primer Name	Forward Primer Sequence<br>Reverse Primer Sequence	Primer position(s)<br>Amplicon size	Non default<br>PCR condition
SLCO2A1-F1<br>*SLCO2A1*-R1	TTCAAGAGCAGCCTCACC<br>TGCCGTAGTCCACAAAGA	EX2-EX5ic<br>559 bp cDNA	Ta:53°C<br>40 cycli
SLCO2A1-F2<br>*SLCO2A1*-R2	GGACCGGCTTTCGGGTA<br>ATCTGGGCACGAGCAGTC	EX5-EX10ic<br>705 bp cDNA	Ta:60°C
SLCO2A1-F3<br>*SLCO2A1*-R3	CACCATCATCACCATCTCCA<br>TGTTCTTCTTCACCCTCCA	EX9-EX14ic<br>689 bp cDNA	Ta:51°C
SLCO2A1-F4<br>*SLCO2A1*-R4	CGACTGGAGCACGAGGACACTGA<br>GAGAGGAAGTGCGGGAGGGTGAG	5'RACE-EX3ic<br>435 bp cDNA (RACE)	
SLCO2A1-F5<br>*SLCO2A1*-R5	GGACACTGACATGGACTGAAGGAGTA<br>GCCAAAGTAGCTGACAAAGATGATGA	5'RACE-EX3ic<br>332 bp cDNA (RACE)	
SLCO2A1-F6<br>*SLCO2A1*-R6	TCAGGGGCAAGTTTCAGC<br>GCTGTCAACGATACGCTACG	EX14-3'RACEic<br>276 bp cDNA (RACE)	
SLCO2A1-F7<br>*SLCO2A1*-R7	AACCCTGGTCCTTCTTGCTG<br>ACGTAACGGCATGACAGTGTTT	EX14-3'RACEic<br>201 bp cDNA (RACE)	
SLCO2A1-F8	CCCGCTCTATCTTCAGCAAC	EX1	
SLCO2A1-F9<br>*SLCO2A1*-R9	TTGACACCGGAGGGAAGGAG<br>CGCAATTACATACGCCAAGCA	PROM-IN1ic<br>521 bp gDNA	Ta:62°C
SLCO2A1-F10<br>*SLCO2A1*-R10	CGGGCGCAAAGAGCAGTTTA<br>CGCCGCCGATGATGAAAT	PROM-PROMic<br>664 bp gDNA	Ta:67°C
SLCO2A1-F11<br>*SLCO2A1*-R11	TGCCTGGAAGTAGCTGGAGAGG<br>GCCGGTCTCTGCTTGTTTGC	PROM-PROMic<br>576 bp gDNA	Ta:63°C
SLCO2A1-R12	GCTTCTCGATGGTGGTG	EX2ic	
SLCO2A1-F13<br>*SLCO2A1*-R13	GCCGTCCTCATCATCTTTGT<br>GAAGTGCGGGAGGGTGA	EX3-EX3ic<br>117 bp cDNA/gDNA	Ta:60°C
SLCO2A1-R14	AGGCGGCTGCTGTTCC	EX4ic	
SLCO2A1-F15<br>*SLCO2A1*-R15	TCCACTGCCCTTTATTCCAG<br>CCTCATCCTCTGCCCTGAT	IN3-IN5ic<br>1334 bp gDNA	
SLCO2A1-F16<br>*SLCO2A1*-R16	AGACCCTCGGTGGATAGGAG<br>CCATCCCTCTGGACATCG	EX6-EX6ic<br>113 bp cDNA/gDNA	Ta:58°C
SLCO2A1-R17	GCGCCATACTGCTTCT	EX8ic	
SLCO2A1-F18<br>*SLCO2A1*-R18	CCTCTCCACCTTCCTCAACA<br>TGATGCCCCACTCACACA	EX8-EX11ic<br>466 bp cDNA	Ta:60°C
SLCO2A1-F19<br>*SLCO2A1*-R19	CCTTGGGGATGCTGTTTG<br>TGGAGATGGTGATGATGGTG	EX9-EX9ic<br>96 bp cDNA/gDNA	Ta:60°C
SLCO2A1-F20	GTGCCCAGATTCCCTCTTC	EX10	

SLCO2A1-F21<br>*SLCO2A1*-R21	GGCCATCTTCCTCATCTCCT<br>GCAAGCGCATCAACAAGAAC	EX11-EX12ic<br>137 bp cDNA	Ta:54°C
SLCO2A1-F22<br>*SLCO2A1*-R22	ACGAGGGCAAGTTCTGATGT<br>TGCTGTCTGTGGTTGTGTTG	IN11-IN12ic<br>283 bp gDNA	Ta:64°C
SLCO2A1-F23<br>*SLCO2A1*-R23	CTGCCATCTCCAGCCCTCT<br>CCTCGCCTCCCCAAGCA	EX13-EX13ic<br>80 bp cDNA/gDNA	
SLCO2A1-F24<br>*SLCO2A1*-R24	GCGGGTGAAGAAGAACAAG<br>CCGTGTCCAGAAGGGTCTA	EX14-EX14ic<br>661 bp cDNA/gDNA	
SLCO2A1-F25<br>*SLCO2A1*-R25	GGCCACTTTGGGAAGTATGG<br>TCTTGCTGTGCATCCCTTG	EX14-EX14ic<br>934 bp cDNA/gDNA	
SLCO2A1-F26<br>*SLCO2A1*-R26	CGGTGGAGGTTCAAAGACAG<br>AAGGTTTAGGAGGGCAACCA	EX14-EX14ic<br>686 bp cDNA/gDNA	
SLCO2A1-F27<br>*SLCO2A1*-R27	TGCCAAGGGTGAAACTGGTC<br>GGGCAGACTCAGGGAAATGC	IN1-IN2ic<br>478 bp gDNA	
SLCO2A1-F28<br>*SLCO2A1*-R28	GGCGAGGATCTCTGGGAAAG<br>AAGCGCAGGAGGATTGAGTG	IN2-IN3ic<br>504 bp gDNA	
SLCO2A1-F29<br>*SLCO2A1*-R29	AGGGTCAGGTCGAGCATT<br>CTGCAACTTCTCAGCTCTGTTT	IN5-IN6ic<br>283 bp gDNA	
SLCO2A1-F30<br>*SLCO2A1*-R30	GGGCCACTAAGGACCAGAAG<br>CCACACCCAATGCCACAC	IN6-IN7ic<br>258 bp gDNA	
SLCO2A1-F31<br>*SLCO2A1*-R31	GGGAGCCTGAGTTGCTGTGT<br>TCCTGTCTGTGTCCGGTTCTG	IN7-IN8ic<br>403 bp gDNA	Ta:57°C<br>+ 2x GC-rich
SLCO2A1-F32<br>*SLCO2A1*-R32	TGCACCCAGACCAGCATGT<br>CAGATGAACAAGGGCAAAGGAGA	IN8-IN9ic<br>337 bp gDNA	Ta:65°C
SLCO2A1-F33<br>*SLCO2A1*-R33	CAAGACAAACAGAGCCGCACA<br>TGAACAGGCGGTGGAGAGAA	IN9-IN10ic<br>381 bp gDNA	
SLCO2A1-F34<br>*SLCO2A1*-R34	GGTGTCTGTTGCCCAAACCA<br>AGCTTCCTGTATCACCCCAAGAAC	IN10-IN11ic<br>479 bp gDNA	
SLCO2A1-F35<br>*SLCO2A1*-R35	GCTCTCTGAACTCTCTTCCTAATGATGC<br>TCTGCTGGGTCTTGTTACCTGGA	IN12-IN13ic<br>347 bp gDNA	Ta:65°C
SLCO2A1-F36<br>*SLCO2A1*-R36	TCACTCTGCCCTGAAAGGA<br>ACAAAAAGGAGATGCCGTGT	IN12-EX14ic<br>1401 bp gDNA	
SLCO2A1-F37<br>*SLCO2A1*-R37	GTACAGCGCCTACTTCA<br>AATACAGAGATGGCAAATAA	EX2-EX5ic<br>510 bp cDNA	Ta:53°C<br>40 cycli
SLCO2A1-F38<br>*SLCO2A1*-R38	ATTTGCCATCTCTGTATTTG<br>CAGCACCAATGAGGAAGT	EX5-Ex8/9ic<br>479 bp cDNA	Ta:56°C
SLCO2A1-F39<br>*SLCO2A1*-R39	AGCAACTGACCTACCTGA<br>CATTGTATTCCTTGTTCTTCT	EX10/11-EX14ic<br>453 bp cDNA	Ta:54°C<br>40 cycli
